# Adaptive Variation Regulates the Expression of the Human *SGK1* Gene in Response to Stress

**DOI:** 10.1371/journal.pgen.1000489

**Published:** 2009-05-22

**Authors:** Francesca Luca, Sonal Kashyap, Catherine Southard, Min Zou, David Witonsky, Anna Di Rienzo, Suzanne D. Conzen

**Affiliations:** 1 Department of Human Genetics, The University of Chicago, Chicago, Illinois, United States of America; 2 Department of Medicine, The University of Chicago, Chicago, Illinois, United States of America; National Institute of Genetics, Japan

## Abstract

The Serum and Glucocorticoid-regulated Kinase1 (*SGK1*) gene is a target of the glucocorticoid receptor (GR) and is central to the stress response in many human tissues. Because environmental stress varies across habitats, we hypothesized that natural selection shaped the geographic distribution of genetic variants regulating the level of *SGK1* expression following GR activation. By combining population genetics and molecular biology methods, we identified a variant (rs9493857) with marked allele frequency differences between populations of African and European ancestry and with a strong correlation between allele frequency and latitude in worldwide population samples. This SNP is located in a GR-binding region upstream of *SGK1* that was identified using a GR ChIP-chip. SNP rs9493857 also lies within a predicted binding site for Oct1, a transcription factor known to cooperate with the GR in the transactivation of target genes. Using ChIP assays, we show that both GR and Oct1 bind to this region and that the ancestral allele at rs9493857 binds the GR-Oct1 complex more efficiently than the derived allele. Finally, using a reporter gene assay, we demonstrate that the ancestral allele is associated with increased glucocorticoid-dependent gene expression when compared to the derived allele. Our results suggest a novel paradigm in which hormonal responsiveness is modulated by sequence variation in the regulatory regions of nuclear receptor target genes. Identifying such functional variants may shed light on the mechanisms underlying inter-individual variation in response to environmental stressors and to hormonal therapy, as well as in the susceptibility to hormone-dependent diseases.

## Author Summary

Susceptibility to many common human diseases including hypertension, heart disease, and the metabolic syndrome is associated with increased neuroendocrine signaling in response to environmental stressors. A key component of the human stress response involves increased systemic glucocorticoid secretion that in turn leads to glucocorticoid receptor (GR) activation. As a result, a variety of GR-expressing cell types undergo gene expression changes, thereby providing an integrated physiological response to stress. The *SGK1* gene is a well-established GR target that promotes cellular homeostasis in response to stress. Here, we use a combination of population genetics and molecular biology approaches to identify an SNP (rs9493857) in a distant *SGK1* GR-binding region with unusually large differences in allele frequency between populations of European and African ancestry. Furthermore, rs9493857 shows a strong correlation between allele frequency and distance from the equator, a pattern consistent with a varying selective advantage across environments. Indeed, the ancestral allele at rs9493857 results in increased GR-binding and glucocorticoid-regulated gene expression, suggesting that an increased stress response (i.e., glucocorticoid responsiveness) was advantageous in ancestral human populations. We speculate that, in modern times, such variation could favor the negative effects of a heightened glucocorticoid response, potentially predisposing individuals to chronic diseases such as metabolic syndrome and hypertension.

## Introduction

Substantial genetic and paleontological evidence supports the idea that humans originated in Sub-Saharan Africa and from there expanded across the globe ([1] and references therein). During this dispersal, human populations encountered and settled into new environments that differed in climate, resource availability, pathogen exposure and other features that can challenge human homeostasis. Additional climatic as well as lifestyle changes, e.g. the retreat of the ice sheet and the agricultural transition, further contributed to the environmental diversity that humans adapted to. Many of these critical adaptations likely occurred at the genetic level through Darwinian selection of beneficial genotypes.

When selective pressures vary across local environments, the geographic distribution of the advantageous genotypes and the resulting phenotypes are expected to follow distinctive patterns that mirror the presence and intensity of the selective pressure. For example, human skin pigmentation and body mass markedly differ across populations and are correlated with UV radiation and temperature, respectively [2],[3]. In genome-wide studies, the analysis of allele frequency differences between populations has identified signals of adaptation in genes playing a role in skin pigmentation, host-pathogen interaction, lactase persistence, etc. [4]–[8]. In addition, genes that play a role in cortisol metabolism, sodium homeostasis, and arterial vessel tone were shown to harbor variants that are strongly correlated with latitude [9],[10]. In these analyses, latitude is considered a proxy for climate; accordingly, these findings were interpreted as evidence for adaptation to heat stress and dehydration. More recently, variation in candidate genes for common metabolic disorders was also shown to be correlated with latitude and a set of climate variables that reflect the impact of cold and heat stress on energy homeostasis [11].

In higher organisms, homeostasis of key physiological processes is achieved through the neuroendocrine response to environmental challenge. This physiological response is in part mediated through the activation of nuclear hormone receptors via a stress-induced ligand (e.g., the adrenally secreted hormone cortisol) and subsequent regulation of target gene expression [12]. Ultimately, nuclear receptors and their associated cofactors, in conjunction with cooperating transcription factors, recognize specific DNA sequences within regulatory regions of genes encoding key physiologic response proteins (for review see [13]). In humans, the stress hormone cortisol mediates gene expression via the GR and, to a lesser extent, the mineralocorticoid receptor (MR). Under conditions of environmental stress, including cold, heat, and dehydration, several stress-associated kinases are activated via rapid post-translational modification, commonly phosphorylation. In contrast, following exposure to physiological stressors, the *SGK1* gene is immediately transcriptionally induced via the ligand-bound GR and MR, and its protein product is then constitutively phosphorylated via endogenous PI3-K activity [14],[15]. The rapid transcriptional induction of SGK1 steady-state levels reflects SGK1's key role in the neuroendocrine response [16],[17]. For example, *SGK1* expression regulates sodium homeostasis in the kidney as well as enhances cell survival following exposure to apoptotic stress such as ultraviolet light and hyperosmolality [14], [18]–[23]. Consistent with a key role in fundamental stress responses, *SGK1* is highly conserved across distantly related species [24]–[26]. At the same time, subtle variation in *SGK1*'s regulatory sequences is hypothesized to alter the threshold of SGK1's hormone-mediated induction, and hence increase or decrease SGK1's ultimate level of activity in response to a given environmental stressor.

Under the assumption that the stress response pathway and, in particular, the *SGK1* gene were targets of local selective pressures, we searched for genetic variants that influence *SGK1* expression in response to stress. To this end, we combined population genetics, comparative genomics and molecular biology approaches to identify variants in candidate regulatory region and then tested them by means of functional assays. We found several variants approximately 30 kb upstream of the transcriptional start site (TSS) of *SGK1* that show unusually large differences in allele frequencies between populations and that are strongly correlated with both latitude and climate variables. One of these variants lies within a predicted binding site for Oct1, a transcription factor known to cooperate with the GR [27]. We show by chromatin immunoprecipitation (ChIP) assays that the ancestral allele of this variant (inferred by comparison to the chimpanzee sequence) results in more efficient binding of the GR-Oct1 complex to this sequence. Furthermore, reporter gene expression assays reveal higher levels of glucocorticoid-dependent transcription from the ancestral allele compared to the derived one (i.e., the allele inferred to have been introduced by mutation because it is not present in chimpanzee).

## Results

### Identification of Candidate Regulatory Variants

We used two approaches to search for signatures of adaptation to local environments in the genomic region surrounding the *SGK1* gene. One was to quantify the difference in allele frequency between pairs of populations by means of the F_ST_ summary statistic [28]. The HapMap Phase II data were used in this analysis [29]. The other approach was to measure the correlation between allele frequencies in a large set of population samples and an environmental variable (e.g., latitude), which was assumed to be a good proxy for the selective pressure [11]. In this analysis, we used the Illumina HumanHap 650Y genotype data from the Human Genome Diversity Project (HGDP) panel [30],[31]. At the genome-wide level, the geographic distribution of allele frequencies is mainly determined by the history of migration and population-specific demographic events. Therefore, to distinguish between the effect of population history alone versus that of natural selection, we compared the geographic distribution of genetic variants in the *SGK1* region to that of variants from large genome-wide data sets.

**Figure 1:**
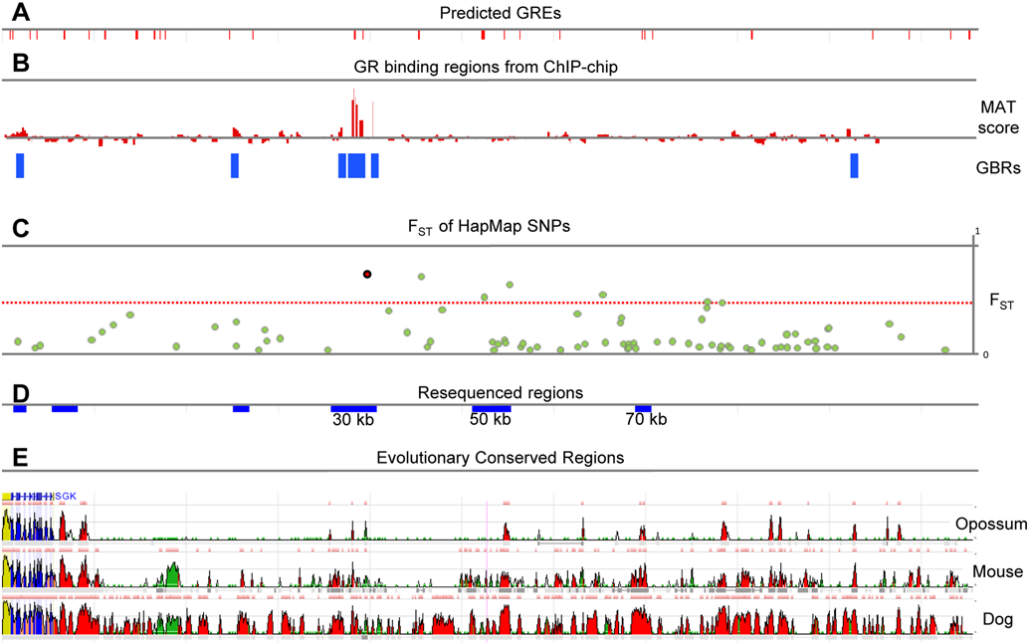
The *SGK1* gene and the genomic region spanning 100 kb upstream of the TSS. (A) Predicted GR binding sites (GREs) identified by NUBIScan. (B) GR-binding sites identified by GR ChIP-chip in MCF10A-Myc cells. The figure shows the MAT score averaged over two independent ChIP-chip experiments. The blue bars identify the sites with a MAT score p-value<10^−3^. (C) F_ST_ value calculated for the Yoruba vs. CEPH European HapMap populations on 82 HapMap Phase II SNPs. The red line indicates the 95th percentile of the FST distribution for all the HapMap Phase II SNPs calculated in the same populations. The SNP marked by a red symbol is rs9493857. (D) Resequenced regions in 14 Italians and 14 Hausa individuals. (E) Evolutionary Conserved Regions (ECR) between Human vs. Opossum, Mouse or Dog obtained from ECR Browser.

We calculated the F_ST_ value between CEPH Europeans and Yoruba for the SNPs in a region of 105.6 kb spanning and upstream of the *SGK1* gene. As shown in Figure 1, the F_ST_ values for seven out of 82 SNPs in this region fall in the top 5% of the empirical distribution for the >2 M HapMap SNPs. In particular, only 0.2% of the HapMap SNPs have an F_ST_ value higher than SNP rs9493857 (shown in red in Figure 1C). These results suggest that the divergence of allele frequency between populations of European and Sub-Saharan African ancestry in the region upstream of *SGK1* is greater than expected based on population history alone.

**Figure 2:**
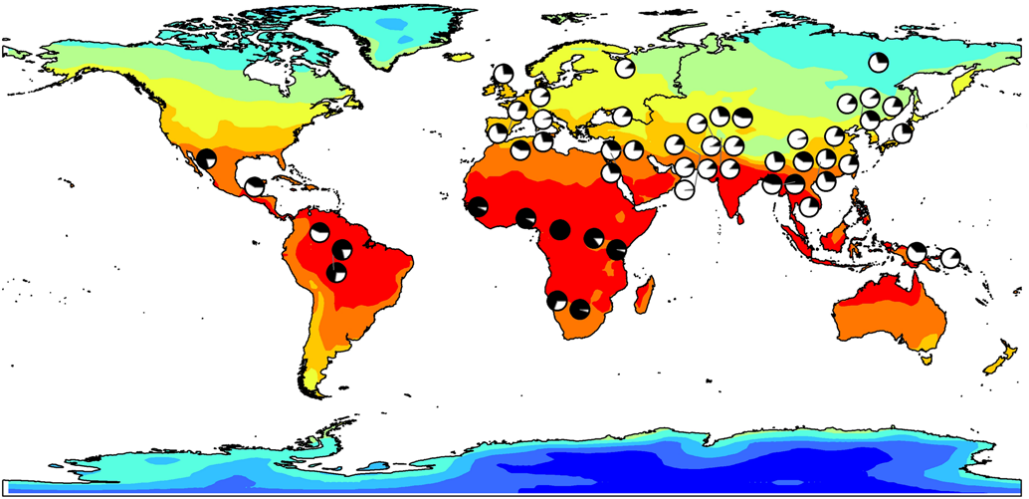
Ancestral allele frequencies for rs9493857 (in black) in the 52 HGDP populations mapped onto a GIS map of Winter Maximum Temperature (matched by hemisphere). Winter Maximum Temperature is the environmental variable with the largest contribution to Winter Principal Component 1.

To test if allele frequencies in the *SGK1* upstream region also correlate with environmental variables, we examined the SNPs genotyped using the Illumina HumanHap 650Y chip in the HGDP panel; in addition, we genotyped two SNPs (rs9493857 and rs1763502) in the same panel. Derived allele frequencies for the 25 SNPs analyzed in the HGDP populations are reported in Table S1. Following the approach described in Hancock et al. (2008) [11], we used two different methods to assess the relationship between allele frequency and environmental variables: Spearman rank correlation and Bayesian geographic analysis. The first one is a non-parametric method that does not assume a linear relationship between the variables. The second one is a model-based method that tests whether a linear relationship between allele frequency and a variable provides a significantly better fit to the data than the null model alone (where the null model is given by a matrix of the covariance of allele frequencies between populations). The environmental variables included latitude and seven climate variables (see Materials and Methods) in the summer and winter seasons; because these variables are partially correlated, we reduced the dimensionality of the data by calculating their principal components and used these new variables to test the correlation with allele frequencies [11]. Eight of the 25 *SGK1* SNPs genotyped in the HGDP panel are significantly (p<0.05) correlated with at least one of the climate principal components or with latitude alone (Table S3). Among them, SNP rs9493857 is the most strongly correlated with latitude and is also significantly correlated with winter Principal Component 1 (Figure 2, Tables S2 and S3). The results of the Bayesian geographic analysis provide more subtle signals, with only three of the 25 *SGK1* SNPs showing a significant linear relationship with the climate principal components (Tables S4 and S5).

We also looked for signatures of natural selection using other aspects of genetic variation data, including the haplotype structure and the allele frequency spectrum [5], [32]–[36]. Unlike the analyses above, these tests did not detect strong signatures of positive selection (Table S7). This may be due to the fact that these tests are known to have inadequate power under a range of selection scenarios; for example, when natural selection acted on recessive variants or on variants present in the population at appreciable frequencies prior to the onset of selection [37],[38].

Overall, the analyses of the geographic distribution of allele frequencies in the region upstream of *SGK1* point to variants that may have been targets of local adaptation. The fact that these candidate selected variants lie in non-coding sequence and that SGK1 activity is primarily regulated by transcriptional induction [14],[15] suggests that these variants may modulate SGK1 activity. However, the only established GR response element (GRE) in this region is located in the *SGK1* promoter [39], while most of the candidate SNPs are located >30 kb upstream of the TSS. To identify additional regulatory regions beyond the promoter, we performed a bioinformatics analysis to identify additional GREs [40] and Evolutionary Conserved Regions (ECRs) between human and dog, mouse, or opossum (Figure 1) [41]. We ultimately considered only regions that contain at least two of the following three features: high FST SNPs, predicted GREs, or ECRs. We thereby narrowed down a region of >100 kb to three candidate regulatory regions (respectively, 30 kb, 50 kb, and 70 kb upstream) spanning a total of 10 kb.

To further prioritize these three candidate regions (and possibly identify additional ones), we performed a GR ChIP-chip assay in MCF10A-Myc mammary epithelial cells treated with the synthetic glucocorticoid (GC) dexamethasone (10^−6^ M). The immunoprecipitated DNA was hybridized onto the Affymetrix GeneChip Human Tiling 2.0R A Array (which covers chromosomes 1 and 6). GR binding regions (GBRs) were then identified using the MAT software [42] to analyze the data obtained from two independent experiments. By using a p-value cutoff of 10^−3^, ChIP-chip identified six GBRs in the *SGK1* region. Among these six GBRs, three nonoverlapping GBRs have a MAT p-value<10^−5^ and also contain two predicted GREs, three ECRs and SNP rs9493857. Of the remaining GBRs, one is located in a region spanning intron 4 to intron 7 and contains a SNP previously implicated in risk to Type II Diabetes and hypertension [43],[44], the second is located 20 kb upstream of the TSS and is close (<1 kb) to a predicted GRE, and the third is located 85 kb upstream of the TSS and is 5 kb away from the closest predicted GRE.

The GBRs close to a predicted GRE as well as the three candidate regulatory regions defined above (30 kb, 50 kb, and 70 kb upstream) were re-sequenced in a panel of 28 individuals (14 Hausa from Cameroon and 14 Italians) to determine whether additional variants with large allele frequency differences between Africans and Europeans exist (see Figure 1). We identified 39 SNPs that were not included in the HapMap data and calculated the F_ST_ values between Hausa and Italians for all SNPs identified by resequencing [45]. As shown in Table S6, rs9493857 retained the highest F_ST_ value among all the SNPs present in the six surveyed regions. Therefore, we hypothesized that rs9493857 is a target of natural selection due to its effect on the induction of *SGK1* expression in response to GR activation. This hypothesis is based on the observation that rs9493857 has both the highest F_ST_ value and the strongest correlation with latitude and that it resides in a GBR.

### Functional Validation of rs9493857 as a Regulatory Variant

**Figure 3:**
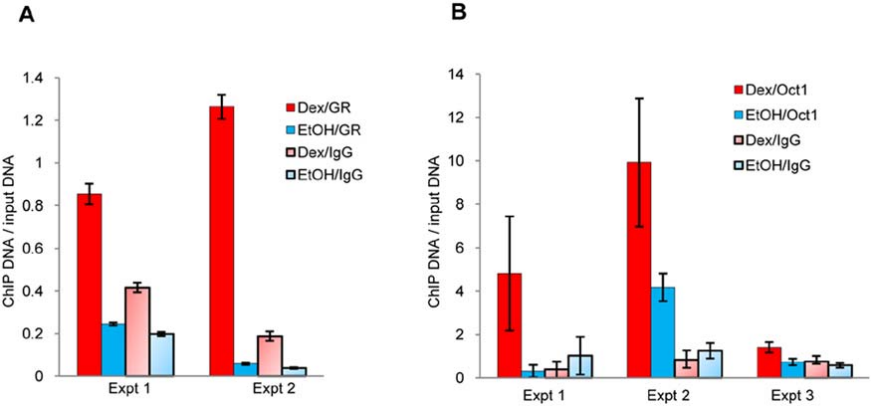
Glucocorticoid-dependent GR and Oct1 occupancy of the region containing rs9493857. (A) GR binds the region containing rs9493857 following dexamethasone (10^−6^ M) treatment in MCF10A-Myc cells. Each measurement is the average of three Q-RT-PCR technical replicates normalized by the input DNA. The error bars represent the standard error. The results of two independent biological replicates are plotted. (B) Oct1 binds the region containing rs9493857 following dexamethasone (10^−6^ M) treatment in MCF10A-Myc cells. Each measurement is the average of three Q-RT-PCR technical replicates normalized by the input DNA. The error bars represent the standard error. The results of three independent biological replicates are plotted.

To validate the results of the GR ChIP-chip assay, we treated MCF10A-Myc breast epithelial cells with either dexamethasone or vehicle and performed a conventional GR-ChIP assay followed by quantitative real-time PCR of the region containing SNP rs9493857. Two independent GR-ChIP experiments showed a significant dexamethasone-dependent enrichment for the region containing rs9493857 (Figure 3A). The results of the conventional ChIP assay allowed us to refine the location of the GBR to a 1 kb region spanning rs9493857. However, this region does not contain a predicted GRE. Therefore, we hypothesized that SNP rs9493857 resides in a binding site for a GR cooperating transcription factor. We used the tool P-Match [46] to search for predicted binding sites for transcription factors and identified a canonical Oct1 binding site that contains rs9493857. Oct1 is a well-established GR cooperating transcription factor that can enhance the regulation of GR target genes in a GC-dependent manner [47]. To confirm that the region containing rs9493857 is indeed an Oct1 binding site, we performed Oct1 ChIP experiments in MCF10A-Myc cells treated with dexamethasone or with vehicle. As shown in Figure 3B, the anti-Oct1 immunoprecipitated chromatin samples were enriched for the region containing rs9493857 in a GC-dependent manner; this enrichment was significant in all three independent experiments (p<0.003, unpaired t-test). In contrast, no enrichment was detected for a negative control region (Figure S1). These results, together with the GR-ChIP results, allowed us to formulate a model in which SNP rs9493857 affects GC-dependent Oct1 occupancy of its predicted binding site, thereby modulating GR-dependent *SGK1* gene expression.

**Figure 4:**
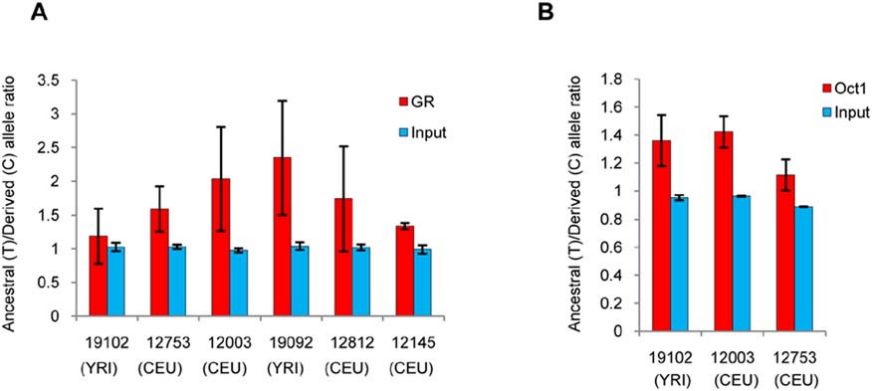
SNP rs9493857 affects GR and Oct1 DNA occupancy following dexamethasone (10^−6^ M) treatment in LCLs. ChIP experiments were performed in HapMap LCLs heterozygous for rs9493857. For each cell line (reported on the horizontal axis), the plot shows the PCR product ratio between the Ancestral and the Derived allele at rs9493857 in the samples immunoprecipitated in the presence of the (A) anti-GR antibody or (B) anti-Oct1 antibody and in the corresponding input samples.

To quantify allele-specific DNA occupancy by the GR-Oct1 complex, we next employed the HaploChIP technique, which allows the direct comparison of two alleles within the same heterozygous sample and the same experiment [48]. Because MCF10A-Myc cells are not heterozygous at SNP rs9493857, we used six lymphoblastoid cell lines (LCLs) from the HapMap project [7] known to be heterozygous at this SNP. Figure 4 shows the results of the GR and Oct1 HaploChIP experiments performed in the presence of dexamethasone. The HaploChIP experiments were performed in duplicate on 6 LCLs for the GR and 3 LCLs for Oct1. As expected, the amount of input DNA (starting material) was equivalent for the two alleles. However, for both GR and Oct1 HaploChIP assays, the amount of immunoprecipitated DNA containing the ancestral allele was significantly greater than that containing the derived allele (p = 0.019 and p = 0.016 for GR and Oct1, respectively). These results support the conclusion that the ancestral allele at rs9493857 results in greater DNA occupancy by the GR-Oct1 complex when compared to the derived allele.

**Figure 5:**
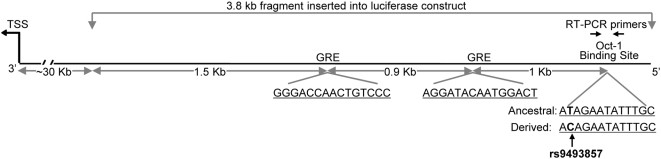
Cartoon of the 3.8 kb enhancer region located 30 kb upstream of the *SGK1* TSS. The entire 3.8 kb enhancer region was used in the reporter gene experiments. The cartoon also shows the predicted GREs, the position of rs9493857 (red) in the predicted Oct1-binding site, and the location of the primers used to evaluate the results of the ChIP experiments.

**Figure 6:**
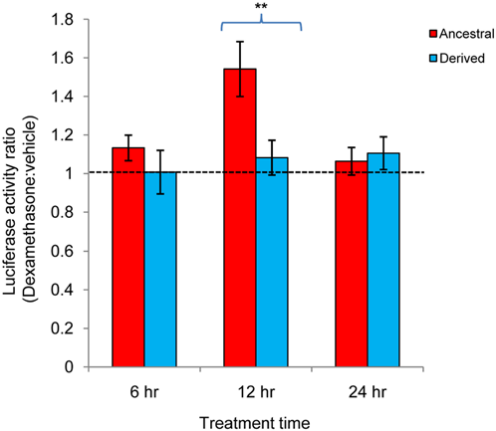
SNP rs9493857 affects glucocorticoid-mediated transcription. SK-BR-3 cells were transiently transfected with a pCMV-ß-galactosidase vector and either the *SGK1* enhancer ANCESTRAL-luciferase or the DERIVED-luciferase reporter plasmid. Cells were split into duplicate plates and treated with either vehicle (EtOH) or dexamethasone (10^−6^ M). Luciferase activity was measured as described previously [91]. The relative luciferase activity in each condition was normalized to ß-galactosidase activity to account for transfection efficiency. Fold change was calculated after normalization to the pGL3 empty vector and reported as an average±standard error of four independent experiments. **, Significant (p = 0.002) one-tailed t-test.

To test whether the newly identified GR-Oct1 binding site is indeed a GC-dependent enhancer region for which the rs9493857 ancestral versus derived alleles convey differential transcriptional activity, we performed luciferase reporter assays in SK-BR-3 breast cancer cells. A 3.8 kb segment encompassing rs9493857 was cloned 5′ to the SV40 promoter driving expression of the luciferase gene (Figure 5). Because SK-BR-3 cells are known to express endogenous GR at relatively high levels [16], the reporter gene assay could be performed with endogenous GR. SK-BR-3 cells were transfected with either the DERIVED-enhancer construct or the ANCESTRAL-enhancer construct, which were identical except for the allele at rs9493857. Upon treatment with dexamethasone for 12 hours, the ancestral allele at rs9493857 resulted in an average of 1.5-fold higher luciferase activity compared to the derived allele (Figure 6) (based on four independent experiments, p = 0.002, one-tailed t-test). These results suggest that the region ∼30 kb upstream of *SGK1* can in fact act as a GC-dependent enhancer whose activity depends upon the particular allele within the Oct1 binding site at rs9493857. In summary, rs9493857 is located within a functional GR enhancer; the ancestral allele at rs9493857 demonstrates both increased GR-Oct1 binding and glucocorticoid-driven gene expression.

## Discussion

We have used a combination of population genetics and molecular biology methods to identify regulatory variants of *SGK1*, a gene that plays a key role in the stress response and that has been clearly implicated in cell survival, water re-absorption and the insulin response. Because *SGK1* expression is induced by the neuroendocrine stress response (through GR and MR activation), we hypothesized that *SGK1* regulatory variation was a target of adaptation to environmental stress. Consistent with this hypothesis, we identified a noncoding variant (rs9493857) with marked allele frequency differences between populations and a strong correlation with climate variables. This SNP is located within a binding site for Oct1, a known GR cooperating transcription factor. Using ChIP-chip, conventional ChIP, HaploChIP and gene reporter assays, we show that the ancestral allele at rs9493857 permits more efficient binding of the GR-Oct1 complex to the enhancer region and induces gene expression at higher levels compared to the derived allele. More broadly, our results show that population genetics approaches may complement computational and traditional molecular biology methods for the identification of regulatory variants in genes involved in the stress response. These variants are expected to contribute to inter-individual differences in hormone (e.g., glucocorticoid) responsiveness, and therefore, could contribute to individual susceptibility to common hormone-dependent diseases, such as cancer and the metabolic syndrome.

Variation in gene regulation has long been hypothesized to be a major mechanism in the phenotypic divergence within and between species [49]–[57]. This proposal was recently bolstered by the genome-wide identification of common variants associated with variation in baseline mRNA levels in LCLs [58],[59]. Consistent with the idea that regulatory variation may contribute to common phenotypes, a large proportion of susceptibility variants for common diseases identified through genome-wide association studies lies in non-coding regions [60]. Moreover, a number of regulatory variants have been shown to be targets of natural selection [61]–[63]. More recently, it was proposed that SNPs showing signals of selection are often associated with variation in baseline expression levels in LCLs, suggesting that selection of gene expression levels plays a key role in human adaptation [64]. However, despite the important role of regulatory variants in health and disease, the identification of such variants continues to present a significant challenge. This is mainly because of the dual challenge in computationally predicting regulatory elements and inferring the functional effects of variation within these elements. To address this problem, two main computational approaches have been developed so far: Prediction of transcription factor binding sites and identification of evolutionarily conserved sequences across distantly related species. Although numerous algorithms have been developed for the computational prediction of transcription factor binding sites, they all suffer from a high false positive discovery rate ([65] and references therein). When overlaying these predictions with sequence conservation, the number of candidate regulatory regions can be narrowed down, but the false positive rate remains too high for experimental follow-up. These two approaches may also be used to predict the effect of genetic variants on gene expression levels, but the accuracy of these predictions remains low.

Many studies aimed at the identification of variation in regulatory sequences have focused exclusively on the proximal promoter region of a gene (generally up to 5–10 kb upstream of the TSS). Consistent with the idea that regulatory variation lies at or near the promoter, genome-wide mapping studies of variation in gene expression found that most expression quantitative trait loci (eQTL) lie in proximity of the TSS [66]. However, it should be noted that these studies were designed to identify eQTLs with strong effects on baseline expression levels, and only limited information is available about the location of eQTLs in response to a physiological stimulus [59],[66],[67]. Therefore, focusing on 5–10 kb upstream of the TSS may miss important regulatory regions, especially for nuclear receptor target genes where long range regulation of gene expression appears to be common [68].

In the present study, we have leveraged both molecular biology and population genetics methods to identify a common variant influencing GR-mediated induction of *SGK1* expression. We used computational predictions of GR binding sites and conserved sequence elements to generate a map of candidate regulatory elements in a >100 kb region encompassing the *SGK1* gene. This map was compared to one generated by GR ChIP-chip analysis. ChIP-chip mapping tends to identify a smaller number of candidate regulatory elements compared to computational methods; however, these regions are still likely to contain a nontrivial portion of false positives [69]. Using the signature of natural selection to prioritize the regions identified by ChIP-chip, we identified a likely GR enhancer region. Moreover, by combining population genetics information with ChIP-chip, we were able to hone in on a regulatory sequence element that harbors common variation in human populations. Although not all variants in GBRs are expected to carry signals of natural selection, this approach is easily amenable to genome-wide applications and may provide testable hypotheses either by itself or in combination with eQTL mapping.

Given the large between-population differences in allele frequency at rs9493857, our results imply that *SGK1* expression levels in response to cortisol could vary greatly across populations with different ancestry. Recent eQTL mapping studies performed on the HapMap LCLs have identified a large fraction of loci with significant differences in mean expression levels among human populations [59]. Although systematic differences between cell lines from different populations may have influenced these results [70], there is clear evidence for inter-population differences in allele frequencies for variants associated with baseline gene expression levels [71],[72]. To investigate the contribution of rs9493857 to *SGK1* mRNA levels, we have inspected the results of genome-wide eQTL studies in which association data are available for all SNPs examined. We did not find a significant association between rs9493857 genotype and *SGK1* mRNA levels [59], [66], [73]–[75]. This finding is not entirely surprising considering that the eQTL studies assayed baseline expression levels while our results indicate that rs9493857 influences gene expression in response to a specific stimulus, i.e. glucocorticoid exposure. Overall, our knowledge of inter-individual variation in expression levels induced by the stress response remains poor.

The results of our functional studies imply that the ancestral allele at rs9493857 will result in higher *SGK1* expression levels in response to physiological stress; this allele is also the most common allele present in populations at lower latitudes. This finding suggests that increased stress-induced *SGK1* gene expression may have been advantageous in ancestral, and perhaps current, human populations living in equatorial environments. Increased *SGK1* expression is consistent with SGK1's role in mediating sodium retention; however, *SGK1* expression is also known to enhance tumor cell survival in breast and prostate cancer cells [14], [18]–[20],[76]. Interestingly, these diverse biological processes (salt retention and breast and prostate cell survival) underlie disease mechanisms with known inter-population differences in incidence. For example, salt-sensitive hypertension and prostate cancer both have a higher prevalence in African Americans compared to other populations [77]–[79]. Similarly, premenopausal African American women have a higher proportion of the subtype of breast cancer known as “triple negative”, namely negative for estrogen, progesterone and Her2 receptors [80]. The lack of these three receptors suggests that alternative growth signaling pathways drive tumor cell proliferation in this breast cancer subtype [81]. Because the PI3-K/SGK1 pathway represents an alternative to ER-, PR- and Her2-mediated growth signaling, increased *SGK1* expression could contribute to susceptibility to triple negative breast cancers [82].

In addition to triple negative breast cancer, African Americans, especially African American women, have a relatively high prevalence of the metabolic syndrome, which includes elevated blood pressure, obesity, and type 2 diabetes [83]. The higher prevalence in African Americans is attributed to both environmental (e.g., diet) and genetic influences. There are many similarities between patients with the metabolic syndrome and those with excessive GC production; however, circulating cortisol levels in the metabolic syndrome are not elevated [84]. This fact suggests that GR signaling may be enhanced in the metabolic syndrome independently of cortisol concentrations. Indeed, SGK1 activity has been associated with hypertension via upregulation of epithelial sodium channel (ENaC) activity. Because small increases in the sodium reabsorptive capacity of the renal epithelia can have dramatic consequences on fluid volume regulation, increased *SGK1* expression might contribute to the development of hypertension [85]. Furthermore, SGK1 activity has been linked to diabetes through glucocorticoid-mediated inhibition of insulin secretion [86]. Interestingly, *SGK1* polymorphisms (located in both Intron 6 and Exon 8) have recently been found to be associated with type 2 diabetes in Romanian and German cohorts [43]. Our finding of a GR-dependent regulatory variant in *SGK1* raises the possibility that inter-individual differences in susceptibility to common diseases may be influenced by differential sensitivities to GR signaling. In other words, individuals harboring alleles resulting in increased cortisol-mediated gene expression may, as a result, be at increased risk of some hormone-dependent diseases such as triple negative breast cancer, prostate cancer, and the metabolic syndrome.

The recent genome-wide association studies potentially offer an opportunity to assess the contribution of SNP rs9493857 to common disease phenotypes. This SNP is not present in the most widely used genotyping platforms, thus only proxy SNPs could be used to analyze the results of genome-wide association studies. None of the proxy SNPs (with r^2^ ranging from 0.8 to 0.6 in Europeans) reaches genome-wide significance in the published studies. However, two of the proxy SNPs, rs4896028 (r^2^ = 0.811) and rs1009840 (r^2^ = 0.616), reach nominal levels of significance for adult BMI (p = 0.027) and glycosylated hemoglobin levels (p = 0.038) (data deposited by WTCCC and published on-line from the British 1958 Birth Cohort DNA Collection, http://www.b58cgene.sgul.ac.uk/), attention deficit hyperactivity disorder (p = 0.01−0.001, as reported in dbGAP), and systemic lupus erythematosus (p = 0.01−0.001, as reported in dbGAP) (Table S8). Further studies are necessary to determine whether SNP rs9493857 indeed influences susceptibility to disease phenotypes. In particular, because this SNP affects glucocorticoid-dependent gene expression, accounting for environmental exposures will be important to determine conclusively if rs9493857 contributes to phenotypic variation related to stress response.

Additional human traits and biological processes that show large inter-population differences include skin pigmentation and energy metabolism [2],[3]. As with the stress response, these processes occur at the interface between the organism and the environment and are important for maintaining homeostasis. Interestingly, in the case of *SGK1*, the target of natural selection appears to be the response to a stress-induced hormonal stimulus. This raises the possibility that a signature of local adaptation, and therefore large inter-population differences, may also be found in a global analysis of genes comprising nuclear receptor gene networks. Further studies are necessary to determine whether additional GR target genes show similar inter-population differences in the frequency of ancestral versus derived regulatory alleles.

## Materials and Methods

### Statistical and Bioinformatics Analyses

GREs were computationally predicted by using NUBIScan 2.0 [40], which implements an algorithm that relies on the combination of nucleotide distribution weight matrices of single hexamer halfsites for the prediction of nuclear receptor response elements. The analysis was performed using the default GR matrix and an arrangement consisting of two inverted repeats spaced by three nucleotides. All the GREs with a raw score ≥0.6 are reported in Figure 1A.

ECRs between human and mouse, dog or opossum were identified using the ECR Browser tool [41].

For each SNP, F_ST_ values between pairs of populations were calculated according to [28]; F_ST_ can vary between 0 and 1, with F_ST_ = 0 indicating no difference in allele frequencies and F_ST_ = 1 indicating that alternative alleles are fixed in the two populations. Spearman rank correlation coefficients between allele frequency and environmental variables were calculated using an in house program. The Bayesian geographic analysis described in Hancock et al. (2008) [11] was applied to the *SGK1* SNPs to assess the evidence for genetic adaptation to varying environments. With both methods, significance was assessed by comparing the value of the test statistic for each SNP to the empirical distribution of the same statistic for the SNPs in the Illumina Infinium HumanHap 650Y chip typed in the HGDP panel [30]. Because a shift in the null distribution was observed for different allele frequency bins and depending upon the genotyping panel used, significance for each *SGK1* SNP was assessed against the distribution of the test statistic for SNPs matched by allele frequency and by panel.

Neutrality tests and summary statistics of genetic variation for the re-sequenced regions were calculated using the program SLIDER (http://genapps.uchicago.edu/slider/index.html). To estimate the significance of Tajima's D and Fay and Wu's H, we performed 1,000 neutral simulations for each population sample separately using the program MS [87]. For the Hausa sample we simulated a simple growth model, while for the Italian sample we simulated a bottleneck model. These demographic scenarios and the corresponding parameter values were chosen based on previous modeling studies showing that they are consistent with patterns of neutral variation in the same population samples [45].

The haplotype test was performed for the re-sequenced candidate regulatory regions as described in [36]. We performed this test separately for each population sample. One thousand replicates were generated under the same demographic scenarios used above.

Re-sequencing of candidate regulatory regions. The DNA samples sequenced at the six candidate regulatory regions belong to a panel previously described [45],[88]. A subset of this panel consisting of 28 unrelated samples (14 Hausa from Cameroon and 14 Italians) was randomly selected.

DNA was PCR amplified and the PCR products, after Exo-SAP purification, were sequenced with ABI BigDye Terminator v. 3.1 Cycle Sequencing kit. The products were analyzed on an ABI 3730 automated sequencer (Applied Biosystems) and the resulting sequences were scored using the software Polyphred version 6.11 [89].

### Cell Lines and Cell Culture

The human breast cancer cell line SK-BR-3 was cultured in DMEM supplemented with 10% FBS and 1% Penicillin/Streptomycin. The human breast epithelial cell line MCF10A-Myc was cultured in DMEM-F12 media supplemented with growth factors as described previously [90]. Six HapMap LCLs heterozygous at SNP rs9493857 were cultured in RPMI supplemented with 15% FBS and 0.1% Gentamicin.

### Plasmid Construction and Luciferase Assays

A 3.8 kb sequence segment upstream of the SGK1 TSS from an Italian individual bearing the derived allele at SNP rs9493857 was cloned in pGL3-Promoter vector (Promega) using the restriction sites KpnI and XhoI. The resulting construct, referred to as “DERIVED,” was subjected to site-directed mutagenesis at the same SNP in order to obtain a construct, referred to as “ANCESTRAL,” which is identical to DERIVED except for the nucleotide at rs9493857. Site-directed mutagenesis was performed according to the manufacturer protocol using the QuikChange II Site-directed Mutagenesis Kit (Stratagene). All constructs were verified by Sanger sequencing and did not contain any artifactual mutations. DNA was prepared using the Qiagen Miniprep and Maxiprep kits and transfected into SK-BR-3 cells using the Polyfect Transfection Reagent (Qiagen). Luciferase and β-galactosidase activity were measured according to standard protocols (Dual-Luciferase Reporter Assay System and Beta-Galactosidase Enzyme Assay, Promega) following 6, 12, and 24 hours of treatment with either 10^−6^ M Dexamethasone or vehicle (ethanol) alone. Results are given as ratios of luciferase over β-galactosidase activity. Four independent experiments were performed and statistical significance between dexamethasone and ethanol treated samples was evaluated by means of a paired one-tailed t-test.

### Conventional ChIP Assays

MCF10A-Myc cells (4–5×10^6^) and LCLs (∼20×10^6^) were serum starved for 48 hours and then treated with dexamethasone 10^−6^ M or ethanol for 1 hour. After treatment, cells were cross-linked for 20 minutes with formaldehyde (1% final concentration) followed by addition of glycine to a final concentration of 125 mM for 5 minutes to arrest the cross-linking. ChIP experiments were performed according to a standard protocol (Upstate Biotechnology, Milipore). Rabbit polyclonal anti-GR (E-20) and anti-Oct1 (C-21) antibodies were obtained from Santa Cruz Laboratories. The immunoprecipitated protein/chromatin complexes were either used to perform a Western-Blot or treated to reverse the crosslinks according to the manufacturer's instructions. Specifically, the Western blot was performed on protein/chromatin complexes obtained from MCF10A-Myc cells to confirm that GR and Oct1 were immunoprecipitated only in the presence of their specific antibodies. The DNA obtained after reversing the crosslinks was either analyzed by quantitative RT-PCR to assess for enrichment of the GR and Oct1 binding regions of interest (DNA from MCF10A-Myc cells) or used to perform the HaploChIP experiments (DNA from LCLs).

### ChIP-Chip Analysis

MCF10A-Myc cells were serum-starved for 48 hours and then treated with dexamethasone (10^−6^ M) for 1 hour. Following standard ChIP, the immunoprecipitated and the input DNA were amplified, fragmented, and labeled for hybridization according to the Affymetrix ChIP Protocol. These samples were then hybridized to the Affymetrix Human Tiling Array 2.0R A (chromosome 1 and 6) and scanned at the University of Chicago Functional Genomics Core Facility. Probe signals from two independent biological experiments were analyzed using the Model-based Analysis of Tiling-array (MAT) software [42] to detect enriched regions of GR binding based on the National Center for Biotechnology Information's build 36 of the human genome. The MAT software identifies ChIP-enriched regions by calculating a MAT score for a given window size. The window size was set to 300 bp based on our observed DNA fragment size after shearing. For each 300 bp sliding window region, a MAT score was calculated by pooling all of the probes across each replicate. To assign a p-value to a window, MAT estimates the non-enriched null distribution of all the MAT scores. To obtain the distribution, MAT uses a non-overlapping sliding window method along the chromosome to calculate MAT scores that cover the array. Assuming MAT scores to be normally distributed, MAT estimates the variance from the windows with MAT scores smaller than the median; the null distribution is then estimated to be symmetric around the median. A threshold of P<10^−3^ was used to identify regions in chromosome 1 and 6 that are occupied by the GR.

### HaploChIP

The DNA obtained by ChIP performed on LCLs treated with dexamethasone was genotyped by means of quantitative RT-PCR using TaqMan reagents. A custom TaqMan genotyping assay was designed to target SNP rs9493857, with the fluorochrome VIC identifying the ancestral allele and FAM identifying the derived allele. To account for differences between the two fluorochromes, a standard curve was built for each of the two alleles using serial dilutions of a genomic DNA known to be heterozygous at rs9493857. The resulting PCR product was quantified for each allele separately in each reaction. The imbalance between the ancestral and the derived alleles was measured as the ratio of the amount of each PCR-product in the immunoprecipitated DNA to that in the corresponding input DNA. Two independent experiments were performed for each cell line and each experiment result was assayed in three RT-PCR technical replicates. Statistical significance was assessed by performing binomial tests on 12 and 6 independent experiments, for the GR and the Oct-1 HaploChIPs, respectively.

### Genotyping Assay

Rs9493857 and rs1763502 were genotyped in 971 individuals from 52 worldwide human populations from the CEPH Human Genome Diversity Project (HGDP) panel [31], using an Illumina GoldenGate assay at the UCLA Southern California Genotyping Consortium Facility.

## Supporting Information

Figure S1 Oct1 does not bind to a negative control region following dexamethasone (10^−6^ M) treatment in MCF10A-Myc cells. Each measurement is the average of three RT-PCR technical replicates normalized to the input DNA. The error bar represents the standard error. The results of two independent biological replicates are plotted.
(3.04 MB TIF)

Table S1 Derived allele frequencies of the *SGK1* SNPs genotyped in the HGDP.
(0.24 MB DOC)

Table S2 Spearman rank correlation coefficients for the *SGK1* SNPs genotyped in the HGDP.
(0.09 MB DOC)

Table S3 Spearman rank correlation coefficients' empirical p values for the *SGK1* SNPs genotyped in the HGDP.
(0.12 MB DOC)

Table S4 Bayes Factors for the *SGK1* SNPs genotyped in the HGDP.
(0.08 MB DOC)

Table S5 Bayes Factors empirical p values for the *SGK1* SNPs genotyped in the HGDP.
(0.09 MB DOC)

Table S6 F_ST_ values in Hausa vs. Italians for the SNPs identified by re-sequencing.
(0.06 MB DOC)

Table S7 Summary statistics for the six regions resequenced in Hausa (A), Italians (B) and the overall (C) sample.
(0.07 MB DOC)

Table S8 Association p values for rs9493857 and proxy SNPs from publicly available datasets.
(0.08 MB DOC)
