# Supplementary figures and images for: Adaptive Variation Regulates the Expression of the Human SGK1 Gene in Response to Stress
Source: PLoS Genet. 2009 May 22;5(5):e1000489. doi: 10.1371/journal.pgen.1000489 (PMC2679193; doi:10.1371/journal.pgen.1000489)

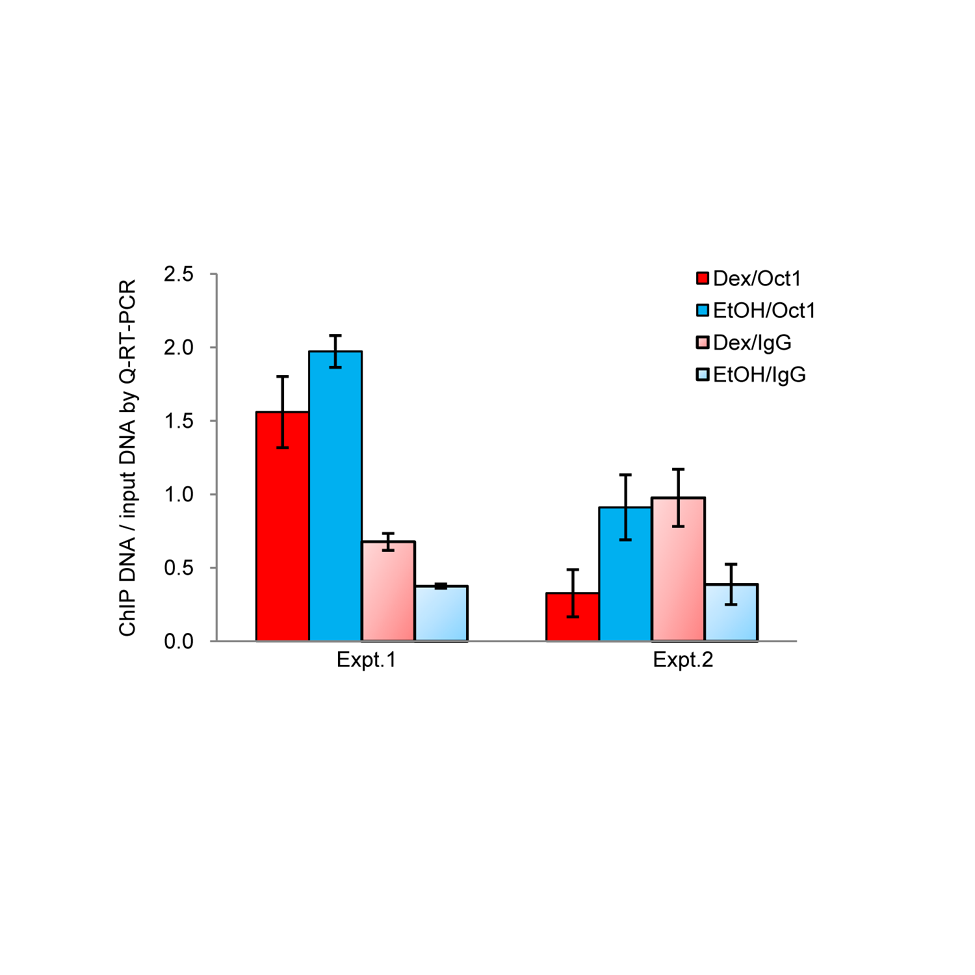

Supplement: Figure S1 — Oct1 does not bind to a negative control region following dexamethasone (10−6 M) treatment in MCF10A-Myc cells. Each measurement is the average of three RT-PCR technical replicates normalized to the input DNA. The error bar represents the standard error. The results of two independent biological replicates are plotted. (3.04 MB TIF) [file pgen.1000489.s001.tif]
