# Supplementary material for: Adaptive Variation Regulates the Expression of the Human SGK1 Gene in Response to Stress
Source: PLoS Genet. 2009 May 22;5(5):e1000489. doi: 10.1371/journal.pgen.1000489 (PMC2679193; doi:10.1371/journal.pgen.1000489)
Supplement: Table S1 — Derived allele frequencies of the SGK1 SNPs genotyped in the HGDP. (0.24 MB DOC) [file pgen.1000489.s002.doc]

Table S1: Derived allele frequencies of the SGK SNPs genotyped in the HGDP

| Country | Population | rs1009840 | rs1114707 | rs17063554 | rs17063563 | rs1743939 | rs1743940 | rs1743955 | rs1763500 | rs1763502 | rs1763509 | rs1763510 | rs1763527 | rs17827161 | rs1981093 | rs4896028 | rs4896032 | rs4896033 | rs4896036 | rs6569934 | rs9373085 | rs9376020 | rs9483670 | rs9493857 | rs9493871 | rs9493873 |
| --- | --- | --- | --- | --- | --- | --- | --- | --- | --- | --- | --- | --- | --- | --- | --- | --- | --- | --- | --- | --- | --- | --- | --- | --- | --- | --- |
| Kenya | Bantu NE | 0.09 | 0.18 | 0.00 | 0.45 | 0.91 | 0.91 | 0.41 | 0.23 | 0.14 | 0.59 | 0.05 | 0.32 | 0.00 | 0.23 | 0.23 | 0.59 | 0.00 | 0.09 | 0.23 | 0.05 | 0.00 | 0.45 | 0.05 | 0.23 | 0.14 |
| Central African Republic | Biaka Pygmy | 0.07 | 0.16 | 0.00 | 0.75 | 0.52 | 0.52 | 0.41 | 0.36 | 0.19 | 0.25 | 0.05 | 0.18 | 0.00 | 0.02 | 0.23 | 0.52 | 0.00 | 0.34 | 0.41 | 0.05 | 0.00 | 0.77 | 0.06 | 0.14 | 0.00 |
| Senegal | Mandenka | 0.16 | 0.32 | 0.00 | 0.89 | 0.70 | 0.70 | 0.25 | 0.25 | 0.27 | 0.20 | 0.14 | 0.34 | 0.05 | 0.30 | 0.32 | 0.57 | 0.00 | 0.25 | 0.57 | 0.11 | 0.05 | 0.52 | 0.15 | 0.30 | 0.00 |
| Democratic Republic of Congo | Mbuti Pygmy | 0.35 | 0.38 | 0.23 | 0.54 | 0.85 | 0.62 | 0.08 | 0.12 | 0.35 | 0.46 | 0.31 | 0.38 | 0.00 | 0.04 | 0.35 | 0.50 | 0.00 | 0.19 | 0.58 | 0.19 | 0.00 | 0.81 | 0.31 | 0.12 | 0.04 |
| Namibia | San | 0.00 | 0.10 | 0.30 | 0.20 | 1.00 | 0.70 | 0.00 | 0.30 | 0 | 0.50 | 0.00 | 0.10 | 0.00 | 0.00 | 0.00 | 0.60 | 0.00 | 0.00 | 0.40 | 0.00 | 0.00 | 0.50 | 0.08 | 0.00 | 0.00 |
| Nigeria | Yoruba | 0.02 | 0.19 | 0.00 | 0.64 | 0.71 | 0.71 | 0.38 | 0.19 | 0.09 | 0.26 | 0.02 | 0.33 | 0.00 | 0.26 | 0.12 | 0.38 | 0.00 | 0.45 | 0.45 | 0.05 | 0.00 | 0.57 | 0.05 | 0.21 | 0.06 |
| S. Africa Bantu | Bantu S. | 0.00 | 0.06 | 0.00 | 0.75 | 0.94 | 0.94 | 0.50 | 0.13 | 0.06 | 0.25 | 0.00 | 0.38 | 0.00 | 0.06 | 0.13 | 0.50 | 0.00 | 0.25 | 0.06 | 0.00 | 0.00 | 0.63 | 0 | 0.38 | 0.00 |
| Russia Caucasus | Adygei | 0.68 | 0.21 | 0.03 | 0.85 | 0.94 | 0.91 | 0.74 | 0.06 | 0.71 | 0.74 | 0.59 | 0.62 | 0.03 | 0.09 | 0.71 | 0.47 | 0.03 | 0.50 | 0.26 | 0.24 | 0.09 | 0.91 | 0.85 | 0.15 | 0.03 |
| France | Basque | 0.54 | 0.35 | 0.02 | 0.83 | 0.92 | 0.90 | 0.60 | 0.06 | 0.56 | 0.73 | 0.58 | 0.50 | 0.00 | 0.04 | 0.56 | 0.44 | 0.02 | 0.54 | 0.40 | 0.35 | 0.21 | 0.94 | 0.71 | 0.17 | 0.02 |
| Italy | Bergamo | 0.63 | 0.29 | 0.00 | 0.83 | 0.79 | 0.79 | 0.63 | 0.13 | 0.69 | 0.71 | 0.54 | 0.46 | 0.00 | 0.08 | 0.67 | 0.38 | 0.04 | 0.63 | 0.38 | 0.25 | 0.17 | 0.96 | 0.88 | 0.04 | 0.04 |
| France | French | 0.75 | 0.25 | 0.05 | 0.89 | 0.95 | 0.89 | 0.71 | 0.04 | 0.75 | 0.75 | 0.71 | 0.59 | 0.05 | 0.09 | 0.75 | 0.32 | 0.04 | 0.63 | 0.29 | 0.23 | 0.21 | 0.89 | 0.82 | 0.13 | 0.04 |
| Orkney Islands | Orcadian | 0.60 | 0.30 | 0.10 | 0.80 | 0.80 | 0.70 | 0.43 | 0.13 | 0.7 | 0.63 | 0.60 | 0.50 | 0.00 | 0.13 | 0.70 | 0.37 | 0.00 | 0.67 | 0.57 | 0.27 | 0.17 | 0.83 | 0.73 | 0.00 | 0.00 |
| Russia | Russian | 0.76 | 0.22 | 0.06 | 0.84 | 0.92 | 0.86 | 0.70 | 0.02 | 0.75 | 0.80 | 0.70 | 0.58 | 0.02 | 0.06 | 0.80 | 0.42 | 0.00 | 0.58 | 0.30 | 0.20 | 0.10 | 0.94 | 0.88 | 0.06 | 0.00 |
| Italy | Sardinian | 0.59 | 0.34 | 0.07 | 0.80 | 0.84 | 0.77 | 0.54 | 0.13 | 0.59 | 0.70 | 0.59 | 0.36 | 0.00 | 0.16 | 0.59 | 0.54 | 0.04 | 0.43 | 0.46 | 0.30 | 0.11 | 0.82 | 0.71 | 0.05 | 0.04 |
| Italy | Tuscan | 1.00 | 0.36 | 0.00 | 1.00 | 1.00 | 1.00 | 0.57 | 0.00 | 1 | 1.00 | 1.00 | 0.64 | 0.14 | 0.07 | 1.00 | 0.36 | 0.00 | 0.64 | 0.43 | 0.36 | 0.29 | 0.93 | 0.94 | 0.00 | 0.00 |
| Israel | Bedouin | 0.60 | 0.50 | 0.02 | 0.84 | 0.86 | 0.83 | 0.46 | 0.14 | 0.61 | 0.72 | 0.62 | 0.38 | 0.01 | 0.11 | 0.62 | 0.43 | 0.02 | 0.52 | 0.53 | 0.44 | 0.16 | 0.83 | 0.69 | 0.10 | 0.02 |
| Israel | Druze | 0.71 | 0.37 | 0.05 | 0.90 | 0.83 | 0.79 | 0.55 | 0.13 | 0.73 | 0.73 | 0.67 | 0.45 | 0.01 | 0.19 | 0.73 | 0.37 | 0.12 | 0.50 | 0.45 | 0.36 | 0.19 | 0.81 | 0.77 | 0.06 | 0.13 |
| Algeria | Mozabite | 0.52 | 0.35 | 0.07 | 0.89 | 0.67 | 0.59 | 0.41 | 0.30 | 0.57 | 0.48 | 0.44 | 0.33 | 0.07 | 0.28 | 0.59 | 0.57 | 0.00 | 0.44 | 0.56 | 0.26 | 0.09 | 0.74 | 0.59 | 0.06 | 0.00 |
| Israel | Palestinian | 0.58 | 0.46 | 0.10 | 0.80 | 0.86 | 0.76 | 0.36 | 0.11 | 0.61 | 0.60 | 0.52 | 0.36 | 0.02 | 0.10 | 0.60 | 0.41 | 0.00 | 0.55 | 0.60 | 0.50 | 0.23 | 0.85 | 0.66 | 0.11 | 0.00 |
| Pakistan | Balochi | 0.83 | 0.31 | 0.00 | 0.85 | 0.96 | 0.96 | 0.73 | 0.02 | 0.85 | 0.88 | 0.73 | 0.54 | 0.02 | 0.04 | 0.85 | 0.44 | 0.04 | 0.54 | 0.27 | 0.27 | 0.08 | 0.96 | 0.98 | 0.08 | 0.04 |
| Pakistan | Brahui | 0.78 | 0.36 | 0.02 | 0.92 | 0.96 | 0.94 | 0.62 | 0.06 | 0.78 | 0.88 | 0.82 | 0.44 | 0.00 | 0.04 | 0.78 | 0.36 | 0.00 | 0.64 | 0.38 | 0.30 | 0.04 | 0.96 | 0.84 | 0.06 | 0.00 |
| Pakistan | Burusho | 0.68 | 0.36 | 0.10 | 0.74 | 0.98 | 0.88 | 0.60 | 0.02 | 0.74 | 0.80 | 0.64 | 0.44 | 0.00 | 0.14 | 0.72 | 0.44 | 0.10 | 0.44 | 0.42 | 0.34 | 0.18 | 0.88 | 0.86 | 0.08 | 0.10 |
| Pakistan | Hazara | 0.70 | 0.41 | 0.09 | 0.80 | 0.95 | 0.86 | 0.39 | 0.05 | 0.74 | 0.86 | 0.73 | 0.45 | 0.05 | 0.11 | 0.73 | 0.41 | 0.05 | 0.57 | 0.64 | 0.43 | 0.34 | 0.80 | 0.85 | 0.00 | 0.05 |
| Pakistan | Kalash | 0.59 | 0.28 | 0.09 | 0.67 | 0.96 | 0.87 | 0.57 | 0.00 | 0.58 | 0.87 | 0.57 | 0.48 | 0.00 | 0.07 | 0.59 | 0.43 | 0.00 | 0.57 | 0.37 | 0.26 | 0.22 | 0.93 | 0.9 | 0.07 | 0.00 |
| Pakistan | Makrani | 0.82 | 0.24 | 0.02 | 0.92 | 0.92 | 0.90 | 0.72 | 0.00 | 0.84 | 0.88 | 0.84 | 0.60 | 0.00 | 0.16 | 0.84 | 0.34 | 0.08 | 0.58 | 0.26 | 0.22 | 0.12 | 0.84 | 0.9 | 0.02 | 0.08 |
| Pakistan | Pathan | 0.77 | 0.50 | 0.02 | 0.84 | 0.98 | 0.95 | 0.43 | 0.00 | 0.81 | 0.89 | 0.75 | 0.27 | 0.02 | 0.09 | 0.84 | 0.41 | 0.05 | 0.55 | 0.57 | 0.50 | 0.27 | 0.89 | 0.9 | 0.09 | 0.05 |
| Pakistan | Sindhi | 0.79 | 0.48 | 0.10 | 0.83 | 0.90 | 0.79 | 0.48 | 0.02 | 0.88 | 0.73 | 0.67 | 0.38 | 0.02 | 0.10 | 0.85 | 0.29 | 0.06 | 0.63 | 0.54 | 0.38 | 0.08 | 0.88 | 0.88 | 0.06 | 0.06 |
| China | Uygur | 0.60 | 0.35 | 0.25 | 0.70 | 1.00 | 0.75 | 0.35 | 0.00 | 0.65 | 0.65 | 0.55 | 0.25 | 0.00 | 0.05 | 0.65 | 0.75 | 0.00 | 0.25 | 0.60 | 0.40 | 0.20 | 0.90 | 0.7 | 0.15 | 0.00 |
| China | Xibo | 0.50 | 0.50 | 0.33 | 0.67 | 0.94 | 0.61 | 0.17 | 0.06 | 0.5 | 0.61 | 0.61 | 0.17 | 0.00 | 0.17 | 0.56 | 0.61 | 0.17 | 0.22 | 0.83 | 0.61 | 0.28 | 0.78 | 0.56 | 0.00 | 0.17 |
| Cambodia | Cambodian | 0.75 | 0.55 | 0.20 | 0.80 | 1.00 | 0.80 | 0.30 | 0.00 | 0.78 | 0.75 | 0.75 | 0.25 | 0.00 | 0.10 | 0.75 | 0.35 | 0.05 | 0.60 | 0.70 | 0.60 | 0.40 | 0.80 | 0.7 | 0.05 | 0.05 |
| China | Dai | 0.55 | 0.55 | 0.45 | 0.55 | 1.00 | 0.55 | 0.15 | 0.00 | 0.55 | 0.55 | 0.55 | 0.15 | 0.00 | 0.05 | 0.55 | 0.40 | 0.05 | 0.55 | 0.90 | 0.55 | 0.30 | 0.75 | 0.55 | 0.00 | 0.05 |
| China | Daur | 0.61 | 0.44 | 0.28 | 0.67 | 1.00 | 0.72 | 0.17 | 0.00 | 0.7 | 0.72 | 0.67 | 0.06 | 0.06 | 0.17 | 0.67 | 0.50 | 0.17 | 0.22 | 0.83 | 0.44 | 0.56 | 0.67 | 0.7 | 0.00 | 0.19 |
| China | Han | 0.76 | 0.60 | 0.24 | 0.73 | 1.00 | 0.76 | 0.18 | 0.00 | 0.78 | 0.75 | 0.72 | 0.15 | 0.00 | 0.07 | 0.78 | 0.50 | 0.05 | 0.43 | 0.83 | 0.59 | 0.52 | 0.82 | 0.81 | 0.01 | 0.05 |
| China | Hezhen | 0.72 | 0.67 | 0.17 | 0.78 | 1.00 | 0.83 | 0.22 | 0.00 | 0.75 | 0.72 | 0.67 | 0.11 | 0.00 | 0.33 | 0.72 | 0.56 | 0.28 | 0.11 | 0.78 | 0.72 | 0.61 | 0.67 | 0.83 | 0.11 | 0.28 |
| Japan | Japanese | 0.70 | 0.54 | 0.36 | 0.63 | 1.00 | 0.64 | 0.30 | 0.00 | 0.71 | 0.64 | 0.63 | 0.23 | 0.00 | 0.18 | 0.68 | 0.38 | 0.16 | 0.46 | 0.70 | 0.50 | 0.46 | 0.68 | 0.71 | 0.00 | 0.16 |
| China | Lahu | 0.50 | 0.44 | 0.44 | 0.56 | 1.00 | 0.56 | 0.19 | 0.00 | 0.5 | 0.50 | 0.50 | 0.13 | 0.00 | 0.19 | 0.50 | 0.69 | 0.19 | 0.13 | 0.81 | 0.38 | 0.44 | 0.88 | 0.5 | 0.06 | 0.19 |
| China | Miaozu | 0.60 | 0.45 | 0.30 | 0.60 | 1.00 | 0.70 | 0.35 | 0.00 | 0.55 | 0.70 | 0.60 | 0.20 | 0.00 | 0.10 | 0.55 | 0.75 | 0.05 | 0.20 | 0.70 | 0.50 | 0.50 | 0.80 | 0.7 | 0.05 | 0.05 |
| China | Mongola | 0.85 | 0.75 | 0.15 | 0.85 | 1.00 | 0.85 | 0.05 | 0.00 | 0.85 | 0.85 | 0.85 | 0.10 | 0.00 | 0.15 | 0.85 | 0.40 | 0.15 | 0.45 | 0.95 | 0.80 | 0.65 | 0.80 | 0.85 | 0.00 | 0.15 |
| China | Naxi | 0.63 | 0.56 | 0.25 | 0.69 | 1.00 | 0.75 | 0.19 | 0.00 | 0.61 | 0.75 | 0.69 | 0.06 | 0.00 | 0.00 | 0.63 | 0.63 | 0.00 | 0.38 | 0.81 | 0.63 | 0.44 | 0.88 | 0.72 | 0.00 | 0.00 |
| China | Oroqen | 0.78 | 0.61 | 0.11 | 0.83 | 1.00 | 0.89 | 0.11 | 0.00 | 0.78 | 0.89 | 0.78 | 0.11 | 0.00 | 0.11 | 0.78 | 0.44 | 0.11 | 0.44 | 0.89 | 0.72 | 0.78 | 0.61 | 0.89 | 0.00 | 0.11 |
| China | She | 0.80 | 0.55 | 0.15 | 0.85 | 1.00 | 0.85 | 0.20 | 0.00 | 0.8 | 0.80 | 0.75 | 0.15 | 0.00 | 0.10 | 0.80 | 0.45 | 0.10 | 0.45 | 0.80 | 0.55 | 0.45 | 0.80 | 0.8 | 0.05 | 0.10 |
| China | Tu | 0.80 | 0.85 | 0.15 | 0.75 | 1.00 | 0.85 | 0.10 | 0.00 | 0.85 | 0.85 | 0.75 | 0.10 | 0.00 | 0.10 | 0.85 | 0.70 | 0.05 | 0.20 | 0.90 | 0.90 | 0.70 | 0.90 | 0.95 | 0.00 | 0.05 |
| China | Tujia | 0.70 | 0.60 | 0.25 | 0.70 | 1.00 | 0.75 | 0.20 | 0.00 | 0.7 | 0.75 | 0.70 | 0.15 | 0.00 | 0.15 | 0.70 | 0.50 | 0.10 | 0.35 | 0.80 | 0.55 | 0.45 | 0.80 | 0.75 | 0.00 | 0.10 |
| Siberia | Yakut | 0.66 | 0.62 | 0.30 | 0.66 | 1.00 | 0.70 | 0.14 | 0.00 | 0.68 | 0.70 | 0.66 | 0.14 | 0.00 | 0.36 | 0.68 | 0.40 | 0.30 | 0.26 | 0.86 | 0.68 | 0.58 | 0.58 | 0.7 | 0.00 | 0.30 |
| China | Yizu | 0.50 | 0.30 | 0.10 | 0.75 | 1.00 | 0.90 | 0.45 | 0.00 | 0.5 | 0.65 | 0.50 | 0.15 | 0.00 | 0.10 | 0.50 | 0.70 | 0.00 | 0.20 | 0.60 | 0.40 | 0.25 | 0.90 | 0.6 | 0.25 | 0.00 |
| Colombia | Piapoco/Curripaco | 0.50 | 0.36 | 0.50 | 0.57 | 1.00 | 0.50 | 0.29 | 0.00 | 0.57 | 0.29 | 0.29 | 0.29 | 0.00 | 0.21 | 0.57 | 0.50 | 0.21 | 0.21 | 0.71 | 0.36 | 0.07 | 0.57 | 0.57 | 0.29 | 0.29 |
| Brazil | Karitiana | 0.23 | 0.46 | 0.77 | 0.23 | 1.00 | 0.23 | 0.08 | 0.00 | 0.21 | 0.15 | 0.15 | 0.19 | 0.00 | 0.38 | 0.23 | 0.58 | 0.38 | 0.00 | 0.92 | 0.69 | 0.08 | 0.58 | 0.21 | 0.08 | 0.38 |
| Mexico | Maya | 0.50 | 0.57 | 0.40 | 0.55 | 0.98 | 0.57 | 0.12 | 0.02 | 0.57 | 0.45 | 0.43 | 0.10 | 0.00 | 0.29 | 0.55 | 0.55 | 0.24 | 0.21 | 0.88 | 0.64 | 0.12 | 0.62 | 0.59 | 0.10 | 0.24 |
| Mexico | Pima | 0.21 | 0.25 | 0.75 | 0.25 | 1.00 | 0.25 | 0.04 | 0.00 | 0.21 | 0.21 | 0.21 | 0.36 | 0.00 | 0.00 | 0.21 | 0.86 | 0.00 | 0.11 | 0.96 | 0.46 | 0.04 | 0.86 | 0.21 | 0.04 | 0.00 |
| Brazil | Surui | 0.19 | 0.19 | 0.81 | 0.19 | 1.00 | 0.19 | 0.00 | 0.00 | 0.28 | 0.19 | 0.19 | 0.13 | 0.00 | 0.13 | 0.19 | 0.31 | 0.13 | 0.56 | 1.00 | 0.31 | 0.19 | 0.31 | 0.28 | 0.00 | 0.13 |
| Bougainville | NAN Melanesian | 0.77 | 0.86 | 0.09 | 0.82 | 0.91 | 0.82 | 0.32 | 0.00 | 0.73 | 0.77 | 0.77 | 0.00 | 0.00 | 0.00 | 0.77 | 0.73 | 0.00 | 0.36 | 0.68 | 0.68 | 0.14 | 0.91 | 0.85 | 0.05 | 0.00 |
| New Guinea | Papuan | 0.56 | 0.85 | 0.09 | 0.59 | 0.68 | 0.59 | 0.09 | 0.00 | 0.62 | 0.56 | 0.56 | 0.00 | 0.00 | 0.00 | 0.62 | 0.38 | 0.00 | 0.53 | 0.91 | 0.82 | 0.09 | 0.91 | 0.62 | 0.03 | 0.00 |
