# Supplementary material for: Adaptive Variation Regulates the Expression of the Human SGK1 Gene in Response to Stress
Source: PLoS Genet. 2009 May 22;5(5):e1000489. doi: 10.1371/journal.pgen.1000489 (PMC2679193; doi:10.1371/journal.pgen.1000489)
Supplement: Table S2 — Spearman rank correlation coefficients for the SGK1 SNPs genotyped in the HGDP. (0.09 MB DOC) [file pgen.1000489.s003.doc]

Table S2: Spearman rank correlation coefficients for the *SGK1* SNPs genotyped in the HGDP (in bold, rs9493857)

| SNP | Latitude | Minimum T (summer) | Minimum T (winter) | Maximum T (summer) | Maximum T (winter) | Mean T (summer) | Mean T (winter) | Precipitation rate (summer) | Precipitation rate (winter) | Short-Wave radiation (summer) | Short-Wave radiation (winter) | Relative humidity (summer) | Relative humidity (winter) | Evaporation potential (summer) | Evaporation potential (winter) | PC1 (winter) | PC2 (winter) | PC1 (summer) | PC2 (summer) |
| --- | --- | --- | --- | --- | --- | --- | --- | --- | --- | --- | --- | --- | --- | --- | --- | --- | --- | --- | --- |
| rs1763527 | 0.232 | -0.213 | -0.022 | 0.081 | -0.063 | 0.004 | -0.009 | -0.551 | 0.166 | 0.404 | -0.155 | -0.383 | 0.026 | 0.402 | -0.050 | 0.093 | 0.093 | 0.477 | 0.079 |
| rs1114707 | 0.101 | 0.095 | -0.170 | -0.110 | -0.241 | -0.022 | -0.216 | 0.298 | -0.026 | -0.125 | -0.207 | 0.198 | 0.149 | -0.210 | -0.260 | 0.179 | 0.092 | -0.168 | -0.125 |
| rs9373085 | 0.148 | 0.029 | -0.218 | -0.154 | -0.278 | -0.072 | -0.266 | 0.322 | -0.042 | -0.110 | -0.259 | 0.206 | 0.145 | -0.224 | -0.260 | 0.233 | 0.067 | -0.163 | -0.190 |
| rs1743955 | -0.272 | 0.155 | 0.096 | -0.026 | 0.148 | 0.010 | 0.081 | 0.485 | -0.143 | -0.440 | 0.195 | 0.347 | -0.119 | -0.327 | 0.182 | -0.163 | -0.117 | -0.477 | -0.067 |
| rs6569934 | -0.069 | 0.124 | -0.029 | -0.135 | -0.039 | -0.053 | -0.066 | 0.501 | -0.034 | -0.392 | -0.046 | 0.410 | 0.017 | -0.370 | 0.009 | 0.018 | -0.006 | -0.421 | -0.190 |
| **rs9493857** | 0.567 | -0.252 | -0.520 | -0.185 | -0.574 | -0.187 | -0.518 | -0.232 | 0.047 | 0.406 | -0.518 | -0.292 | 0.303 | 0.164 | -0.664 | 0.543 | 0.163 | 0.434 | -0.190 |
| rs4896028 | 0.496 | -0.099 | -0.430 | -0.031 | -0.477 | -0.034 | -0.429 | -0.162 | -0.060 | 0.288 | -0.468 | -0.254 | 0.214 | 0.189 | -0.470 | 0.463 | 0.108 | 0.292 | -0.082 |
| rs1763502 | 0.472 | -0.057 | -0.412 | -0.024 | -0.460 | -0.013 | -0.409 | -0.123 | -0.045 | 0.264 | -0.443 | -0.224 | 0.223 | 0.148 | -0.551 | 0.434 | 0.064 | 0.277 | -0.039 |
| rs1763500 | 0.095 | 0.003 | -0.273 | -0.254 | -0.283 | -0.191 | -0.296 | 0.494 | 0.054 | -0.223 | -0.186 | 0.331 | 0.183 | -0.298 | -0.162 | 0.228 | 0.122 | -0.267 | -0.339 |
| rs1009840 | 0.482 | -0.068 | -0.443 | -0.035 | -0.487 | -0.023 | -0.439 | -0.114 | -0.069 | 0.265 | -0.476 | -0.203 | 0.221 | 0.168 | -0.456 | 0.469 | 0.099 | 0.256 | -0.090 |
| rs1763509 | 0.497 | -0.267 | -0.543 | -0.124 | -0.558 | -0.171 | -0.536 | -0.183 | -0.023 | 0.413 | -0.435 | -0.294 | 0.252 | 0.228 | -0.461 | 0.526 | 0.119 | 0.407 | -0.172 |
| rs1763510 | 0.469 | -0.100 | -0.449 | -0.062 | -0.494 | -0.060 | -0.451 | -0.065 | -0.054 | 0.233 | -0.468 | -0.175 | 0.226 | 0.134 | -0.459 | 0.470 | 0.111 | 0.237 | -0.117 |
| rs17827161 | 0.260 | -0.060 | -0.074 | 0.271 | -0.083 | 0.168 | -0.094 | -0.334 | -0.019 | 0.361 | -0.207 | -0.323 | 0.082 | 0.517 | -0.079 | 0.106 | 0.025 | 0.316 | 0.213 |
| rs9376020 | 0.536 | -0.300 | -0.569 | -0.433 | -0.648 | -0.428 | -0.636 | 0.275 | -0.115 | -0.005 | -0.565 | 0.222 | 0.296 | -0.113 | -0.376 | 0.641 | 0.101 | 0.040 | -0.537 |
| rs17063554 | -0.074 | 0.071 | -0.030 | -0.171 | -0.041 | -0.099 | -0.057 | 0.387 | -0.039 | -0.326 | -0.026 | 0.372 | -0.039 | -0.349 | 0.055 | 0.036 | -0.031 | -0.337 | -0.247 |
| rs1743940 | 0.362 | -0.290 | -0.399 | 0.009 | -0.376 | -0.104 | -0.376 | -0.302 | -0.126 | 0.495 | -0.245 | -0.405 | 0.151 | 0.360 | -0.303 | 0.367 | -0.017 | 0.461 | -0.015 |
| rs1743939 | 0.137 | -0.101 | -0.370 | -0.203 | -0.302 | -0.216 | -0.378 | 0.446 | -0.214 | -0.165 | -0.164 | 0.283 | 0.006 | -0.203 | -0.031 | 0.304 | -0.137 | -0.198 | -0.352 |
| rs17063563 | 0.411 | -0.040 | -0.208 | 0.187 | -0.229 | 0.150 | -0.201 | -0.404 | -0.124 | 0.411 | -0.348 | -0.399 | 0.100 | 0.407 | -0.298 | 0.263 | 0.008 | 0.422 | 0.176 |
| rs9493871 | -0.299 | 0.068 | 0.286 | 0.164 | 0.305 | 0.120 | 0.294 | -0.062 | 0.136 | 0.000 | 0.311 | -0.042 | -0.007 | 0.020 | 0.180 | -0.321 | 0.024 | 0.010 | 0.204 |
| rs4896032 | -0.182 | -0.052 | -0.063 | -0.088 | 0.018 | -0.084 | -0.055 | 0.181 | -0.191 | -0.069 | 0.150 | 0.041 | -0.140 | -0.178 | 0.080 | -0.047 | -0.166 | -0.104 | -0.061 |
| rs9493873 | 0.025 | 0.090 | -0.077 | -0.100 | -0.140 | -0.084 | -0.099 | 0.251 | -0.061 | -0.235 | -0.105 | 0.271 | 0.118 | -0.187 | -0.058 | 0.124 | -0.019 | -0.218 | -0.178 |
| rs4896033 | 0.171 | 0.092 | -0.170 | -0.101 | -0.240 | -0.063 | -0.206 | 0.220 | -0.077 | -0.141 | -0.253 | 0.230 | 0.133 | -0.170 | -0.173 | 0.237 | 0.004 | -0.143 | -0.195 |
| rs1981093 | -0.091 | -0.086 | -0.010 | 0.048 | 0.043 | 0.033 | -0.007 | 0.001 | 0.146 | 0.112 | 0.096 | -0.148 | 0.026 | 0.101 | 0.046 | -0.079 | 0.125 | 0.090 | 0.080 |
| rs4896036 | 0.296 | -0.030 | -0.019 | 0.053 | -0.106 | 0.053 | -0.026 | -0.281 | 0.238 | 0.230 | -0.252 | -0.173 | 0.153 | 0.256 | -0.164 | 0.138 | 0.205 | 0.263 | 0.049 |
| rs9483670 | 0.316 | -0.283 | -0.259 | -0.171 | -0.308 | -0.145 | -0.267 | -0.260 | 0.176 | 0.403 | -0.279 | -0.286 | 0.175 | 0.119 | -0.343 | 0.274 | 0.193 | 0.417 | -0.131 |
