# Supplementary material for: Adaptive Variation Regulates the Expression of the Human SGK1 Gene in Response to Stress
Source: PLoS Genet. 2009 May 22;5(5):e1000489. doi: 10.1371/journal.pgen.1000489 (PMC2679193; doi:10.1371/journal.pgen.1000489)
Supplement: Table S4 — Bayes Factors for the SGK1 SNPs genotyped in the HGDP. (0.08 MB DOC) [file pgen.1000489.s005.doc]

Table S4: Bayes Factors for the *SGK1* SNPs genotyped in the HGDP (in bold, rs9493857)

| SNP | Latitude | Minimum T (summer) | Minimum T (winter) | Maximum T (summer) | Maximum T (winter) | Mean T (summer) | Mean T (winter) | Precipitation rate (summer) | Precipitation rate (winter) | Short-Wave radiation (summer) | Short-Wave radiation (winter) | Relative humidity (summer) | Relative humidity (winter) | Evaporation potential (summer) | Evaporation potential (winter) | PC1 (winter) | PC2 (winter) |
| --- | --- | --- | --- | --- | --- | --- | --- | --- | --- | --- | --- | --- | --- | --- | --- | --- | --- |
| rs1763527 | 2.2E-01 | 1.6E-01 | 1.7E-01 | 1.5E-01 | 1.9E-01 | 1.4E-01 | 1.8E-01 | 2.3E-01 | 1.7E-01 | 2.2E-01 | 2.1E-01 | 1.5E-01 | 1.5E-01 | 1.8E-01 | 1.6E-01 | 1.9E-01 | 1.5E-01 |
| rs1114707 | 5.4E-01 | 3.0E-01 | 3.3E-01 | 1.9E-01 | 1.9E-01 | 2.1E-01 | 2.2E-01 | 1.9E-01 | 2.1E-01 | 1.9E-01 | 2.2E-01 | 1.9E-01 | 1.4E-01 | 3.0E-01 | 1.7E-01 | 3.2E-01 | 2.2E-01 |
| rs9373085 | 2.3E-01 | 1.9E-01 | 2.2E-01 | 1.6E-01 | 3.0E-01 | 1.9E-01 | 2.4E-01 | 5.4E-01 | 1.8E-01 | 5.3E-01 | 1.9E-01 | 2.5E-01 | 1.5E-01 | 2.1E-01 | 1.6E-01 | 2.5E-01 | 1.8E-01 |
| rs1743955 | 2.5E-01 | 1.7E-01 | 3.8E-01 | 1.3E-01 | 4.5E-01 | 1.3E-01 | 4.2E-01 | 5.2E-01 | 3.5E-01 | 4.2E-01 | 1.9E-01 | 1.8E-01 | 2.2E-01 | 3.4E-01 | 3.1E-01 | 2.5E-01 | 1.3E-01 |
| rs6569934 | 2.0E-01 | 1.7E-01 | 1.5E-01 | 1.5E-01 | 1.8E-01 | 1.6E-01 | 1.6E-01 | 2.2E-01 | 1.9E-01 | 1.6E-01 | 1.8E-01 | 1.4E-01 | 2.0E-01 | 1.8E-01 | 1.9E-01 | 1.9E-01 | 1.5E-01 |
| **rs9493857** | 3.7E-01 | 1.7E-01 | 4.5E-01 | 2.0E-01 | 6.2E-01 | 2.6E-01 | 4.4E-01 | 1.9E-01 | 3.9E-01 | 2.2E-01 | 3.2E-01 | 2.6E-01 | 1.5E-01 | 4.4E-01 | 1.9E-01 | 2.2E-01 | 2.0E-01 |
| rs4896028 | 2.5E-01 | 3.2E-01 | 2.0E-01 | 1.2E+01 | 2.0E-01 | 3.1E+00 | 1.9E-01 | 2.2E-01 | 1.8E-01 | 2.0E-01 | 2.1E-01 | 2.9E-01 | 1.9E-01 | 2.1E-01 | 1.7E-01 | 2.1E-01 | 2.2E+00 |
| rs1763502 | 2.7E-01 | 3.7E-01 | 2.0E-01 | 4.1E-01 | 2.3E-01 | 5.6E-01 | 1.9E-01 | 2.1E-01 | 1.8E-01 | 2.3E-01 | 3.3E-01 | 2.0E-01 | 1.9E-01 | 2.3E-01 | 1.8E-01 | 3.8E-01 | 3.8E-01 |
| rs1763500 | 1.9E-01 | 1.0E-01 | 1.7E-01 | 1.0E-01 | 1.8E-01 | 9.7E-02 | 1.7E-01 | 1.3E+00 | 2.6E-01 | 3.9E-01 | 1.8E-01 | 3.6E-01 | 1.6E-01 | 1.9E-01 | 1.7E-01 | 5.0E-01 | 1.1E-01 |
| rs1009840 | 2.1E-01 | 4.6E-01 | 1.8E-01 | 7.8E-01 | 2.0E-01 | 1.1E+00 | 1.7E-01 | 1.7E-01 | 2.1E-01 | 1.8E-01 | 2.1E-01 | 2.0E-01 | 1.4E-01 | 1.9E-01 | 1.6E-01 | 2.1E-01 | 6.2E-01 |
| rs1763509 | 3.0E-01 | 2.0E-01 | 6.9E-01 | 1.3E-01 | 9.2E-01 | 1.3E-01 | 7.1E-01 | 3.0E-01 | 1.8E-01 | 4.5E-01 | 2.1E-01 | 2.4E-01 | 2.8E-01 | 6.2E-01 | 1.8E-01 | 5.5E-01 | 1.3E-01 |
| rs1763510 | 2.3E-01 | 3.5E-01 | 2.3E-01 | 5.1E-01 | 2.8E-01 | 7.5E-01 | 2.3E-01 | 2.0E-01 | 2.5E-01 | 1.8E-01 | 2.1E-01 | 2.3E-01 | 1.4E-01 | 2.5E-01 | 1.9E-01 | 2.2E-01 | 4.5E-01 |
| rs17827161 | 2.6E-01 | 1.5E-01 | 2.3E-01 | 1.9E-01 | 2.2E-01 | 1.5E-01 | 2.3E-01 | 2.0E-01 | 1.9E-01 | 1.5E-01 | 2.5E-01 | 1.3E-01 | 1.6E-01 | 2.6E-01 | 1.5E-01 | 1.8E-01 | 1.4E-01 |
| rs9376020 | 2.4E+00 | 6.7E-01 | 4.4E+00 | 6.1E-01 | 2.0E+02 | 9.8E-01 | 2.2E+01 | 2.3E-01 | 1.8E-01 | 1.6E-01 | 1.7E+00 | 1.3E+00 | 3.7E-01 | 1.3E+01 | 1.8E-01 | 1.7E-01 | 1.4E+00 |
| rs17063554 | 2.2E-01 | 1.4E-01 | 2.2E-01 | 1.2E-01 | 2.6E-01 | 1.2E-01 | 2.3E-01 | 2.5E-01 | 1.6E-01 | 1.7E-01 | 1.7E-01 | 1.3E-01 | 2.3E-01 | 2.4E-01 | 1.3E-01 | 1.9E-01 | 1.2E-01 |
| rs1743940 | 4.0E-01 | 2.2E-01 | 8.9E-01 | 1.1E-01 | 5.8E-01 | 1.1E-01 | 7.5E-01 | 1.7E-01 | 1.9E-01 | 2.9E-01 | 1.8E-01 | 1.8E-01 | 1.7E-01 | 7.0E-01 | 1.3E-01 | 2.7E-01 | 1.2E-01 |
| rs1743939 | 3.2E-01 | 1.2E-01 | 4.8E-01 | 1.1E-01 | 3.1E-01 | 1.1E-01 | 3.8E-01 | 2.5E-01 | 2.5E-01 | 1.7E-01 | 1.5E-01 | 1.3E-01 | 1.3E-01 | 3.7E-01 | 1.5E-01 | 1.7E-01 | 1.2E-01 |
| rs17063563 | 2.8E-01 | 1.0E+00 | 2.1E-01 | 1.2E+00 | 2.9E-01 | 1.5E+00 | 2.3E-01 | 1.8E-01 | 4.8E-01 | 1.7E-01 | 1.8E-01 | 4.6E-01 | 3.9E-01 | 2.5E-01 | 6.4E-01 | 2.2E-01 | 9.0E-01 |
| rs9493871 | 9.8E-01 | 1.1E-01 | 2.2E-01 | 1.1E-01 | 2.4E-01 | 1.1E-01 | 2.1E-01 | 3.3E+00 | 2.9E-01 | 2.4E-01 | 4.0E-01 | 2.6E-01 | 2.4E-01 | 3.1E-01 | 2.3E-01 | 7.8E-01 | 1.2E-01 |
| rs4896032 | 2.4E-01 | 1.1E+00 | 2.5E-01 | 1.5E-01 | 2.0E-01 | 1.7E-01 | 2.3E-01 | 3.5E-01 | 2.1E-01 | 1.5E+00 | 1.9E-01 | 1.2E+00 | 2.0E-01 | 2.1E-01 | 2.0E-01 | 2.6E+00 | 1.5E-01 |
| rs9493873 | 1.8E-01 | 1.3E-01 | 2.3E-01 | 1.7E-01 | 6.3E-01 | 1.5E-01 | 3.1E-01 | 1.6E-01 | 1.3E-01 | 1.6E-01 | 5.2E-01 | 3.3E-01 | 4.5E-01 | 4.2E-01 | 2.3E-01 | 1.5E-01 | 1.6E-01 |
| rs4896033 | 2.1E-01 | 1.1E-01 | 2.4E-01 | 1.1E-01 | 4.0E-01 | 1.0E-01 | 2.9E-01 | 2.2E-01 | 2.2E-01 | 1.8E-01 | 2.5E-01 | 2.7E-01 | 1.4E-01 | 3.8E-01 | 1.4E-01 | 1.7E-01 | 1.1E-01 |
| rs1981093 | 3.8E-01 | 1.2E-01 | 2.0E-01 | 1.4E-01 | 3.0E-01 | 1.2E-01 | 2.2E-01 | 1.8E-01 | 4.6E-01 | 2.0E-01 | 4.0E-01 | 3.3E-01 | 1.2E-01 | 3.2E-01 | 1.5E-01 | 2.2E-01 | 1.4E-01 |
| rs4896036 | 4.2E-01 | 6.5E-01 | 4.1E+00 | 2.1E-01 | 1.1E+00 | 3.3E-01 | 2.0E+00 | 1.8E+00 | 4.6E-01 | 4.1E+00 | 2.3E-01 | 2.0E+00 | 1.6E-01 | 1.1E+00 | 2.6E-01 | 4.2E+00 | 2.2E-01 |
| rs9483670 | 2.3E-01 | 1.6E-01 | 1.7E-01 | 1.3E-01 | 1.8E-01 | 1.3E-01 | 1.7E-01 | 2.7E-01 | 3.6E-01 | 1.7E-01 | 2.0E-01 | 2.8E-01 | 1.5E-01 | 1.9E-01 | 1.9E-01 | 2.2E-01 | 1.3E-01 |
