# Supplementary material for: Adaptive Variation Regulates the Expression of the Human SGK1 Gene in Response to Stress
Source: PLoS Genet. 2009 May 22;5(5):e1000489. doi: 10.1371/journal.pgen.1000489 (PMC2679193; doi:10.1371/journal.pgen.1000489)
Supplement: Table S5 — Bayes Factors empirical p values for the SGK1 SNPs genotyped in the HGDP. (0.09 MB DOC) [file pgen.1000489.s006.doc]

Table S5: Bayes Factors empirical p values for the *SGK1* SNPs genotyped in the HGDP (in bold, p<0.05 and rs9493857). The p-values are not corrected for multiple testing.

| SNP | Latitude | Minimum T (summer) | Minimum T (winter) | Maximum T (summer) | Maximum T (winter) | Mean T (summer) | Mean T (winter) | Precipitation rate (summer) | Precipitation rate (winter) | Short-Wave radiation (summer) | Short-Wave radiation (winter) | Relative humidity (summer) | Relative humidity (winter) | PC1 (winter) | PC2 (winter) | PC1 (summer) | PC2 (summer) |
| --- | --- | --- | --- | --- | --- | --- | --- | --- | --- | --- | --- | --- | --- | --- | --- | --- | --- |
| rs1763527 | 0.88 | 0.68 | 0.99 | 0.76 | 0.94 | 0.80 | 0.97 | 0.69 | 0.92 | 0.63 | 0.69 | 0.96 | 0.83 | 0.99 | 0.84 | 0.94 | 0.77 |
| rs1114707 | 0.32 | 0.29 | 0.44 | 0.50 | 0.92 | 0.45 | 0.70 | 0.94 | 0.68 | 0.84 | 0.66 | 0.69 | 0.90 | 0.53 | 0.76 | 0.40 | 0.46 |
| rs9373085 | 0.82 | 0.54 | 0.70 | 0.68 | 0.49 | 0.49 | 0.61 | 0.21 | 0.91 | 0.22 | 0.82 | 0.44 | 0.78 | 0.81 | 0.85 | 0.58 | 0.59 |
| rs1743955 | 0.70 | 0.62 | 0.36 | 0.98 | 0.32 | 0.95 | 0.33 | 0.22 | 0.33 | 0.27 | 0.80 | 0.70 | 0.40 | 0.45 | 0.33 | 0.58 | 0.99 |
| rs6569934 | 0.92 | 0.61 | 1.00 | 0.77 | 0.91 | 0.63 | 0.97 | 0.76 | 0.80 | 0.99 | 0.87 | 0.99 | 0.52 | 0.94 | 0.66 | 0.94 | 0.74 |
| **rs9493857** | 0.44 | 0.64 | 0.30 | 0.45 | 0.24 | 0.33 | 0.31 | 0.94 | 0.30 | 0.63 | 0.40 | 0.41 | 0.78 | 0.34 | 0.60 | 0.72 | 0.50 |
| rs4896028 | 0.70 | 0.25 | 0.78 | **0.01** | 0.80 | **0.03** | 0.86 | 0.75 | 0.91 | 0.74 | 0.69 | 0.36 | 0.51 | 0.86 | 0.73 | 0.79 | **0.04** |
| rs1763502 | 0.63 | 0.22 | 0.80 | 0.20 | 0.65 | 0.14 | 0.85 | 0.81 | 0.90 | 0.58 | 0.39 | 0.60 | 0.52 | 0.70 | 0.66 | 0.33 | 0.24 |
| rs1763500 | 0.83 | 0.99 | 0.77 | 0.96 | 0.71 | 0.99 | 0.76 | **0.04** | 0.31 | 0.19 | 0.65 | 0.15 | 0.48 | 0.71 | 0.51 | 0.15 | 0.85 |
| rs1009840 | 0.96 | 0.17 | 0.99 | 0.10 | 0.90 | 0.08 | 0.99 | 1.00 | 0.69 | 0.89 | 0.70 | 0.60 | 0.94 | 0.96 | 0.88 | 0.77 | 0.15 |
| rs1763509 | 0.56 | 0.48 | 0.20 | 0.97 | 0.17 | 0.98 | 0.20 | 0.42 | 0.91 | 0.25 | 0.69 | 0.45 | 0.31 | 0.25 | 0.66 | 0.21 | 0.98 |
| rs1763510 | 0.83 | 0.24 | 0.66 | 0.16 | 0.54 | 0.11 | 0.66 | 0.92 | 0.52 | 0.93 | 0.70 | 0.52 | 0.90 | 0.66 | 0.60 | 0.77 | 0.20 |
| rs17827161 | 0.53 | 0.56 | 0.48 | 0.31 | 0.57 | 0.48 | 0.53 | 0.72 | 0.61 | 0.94 | 0.42 | 0.79 | 0.54 | 0.47 | 0.65 | 0.84 | 0.54 |
| rs9376020 | 0.09 | 0.10 | 0.06 | 0.12 | **0.01** | 0.07 | **0.02** | 0.61 | 0.83 | 0.97 | 0.10 | 0.09 | 0.23 | **0.03** | 0.67 | 0.97 | 0.06 |
| rs17063554 | 0.73 | 0.74 | 0.52 | 0.92 | 0.46 | 0.81 | 0.50 | 0.46 | 0.94 | 0.86 | 0.87 | 0.97 | 0.31 | 0.52 | 0.98 | 0.76 | 0.95 |
| rs1743940 | 0.38 | 0.37 | 0.14 | 1.00 | 0.23 | 1.00 | 0.17 | 0.98 | 0.77 | 0.40 | 0.86 | 0.63 | 0.60 | 0.20 | 1.00 | 0.48 | 1.00 |
| rs1743939 | 0.41 | 0.77 | 0.16 | 0.77 | 0.34 | 0.80 | 0.24 | 0.42 | 0.36 | 0.80 | 0.86 | 0.83 | 0.82 | 0.28 | 0.66 | 0.92 | 0.71 |
| rs17063563 | 0.56 | 0.06 | 0.65 | 0.07 | 0.45 | **0.05** | 0.57 | 0.94 | 0.23 | 0.94 | 0.75 | 0.23 | 0.23 | 0.58 | 0.17 | 0.67 | 0.09 |
| rs9493871 | 0.09 | 0.93 | 0.46 | 0.74 | 0.42 | 0.82 | 0.50 | **0.01** | 0.26 | 0.38 | 0.19 | 0.23 | 0.23 | 0.31 | 0.28 | 0.08 | 0.70 |
| rs4896032 | 0.77 | 0.06 | 0.60 | 0.73 | 0.82 | 0.60 | 0.66 | 0.36 | 0.67 | 0.10 | 0.77 | 0.11 | 0.51 | 0.81 | 0.59 | 0.06 | 0.86 |
| rs9493873 | 0.92 | 0.85 | 0.45 | 0.44 | 0.08 | 0.66 | 0.22 | 0.95 | 1.00 | 0.87 | 0.08 | 0.16 | 0.09 | 0.14 | 0.32 | 0.99 | 0.57 |
| rs4896033 | 0.79 | 0.85 | 0.45 | 0.76 | 0.23 | 0.88 | 0.34 | 0.59 | 0.42 | 0.70 | 0.42 | 0.22 | 0.64 | 0.26 | 0.79 | 0.88 | 0.80 |
| rs1981093 | 0.35 | 0.92 | 0.56 | 0.59 | 0.38 | 0.77 | 0.51 | 0.76 | 0.22 | 0.56 | 0.29 | 0.25 | 0.90 | 0.38 | 0.71 | 0.58 | 0.62 |
| rs4896036 | 0.40 | 0.12 | 0.06 | 0.45 | 0.16 | 0.26 | 0.09 | 0.06 | 0.25 | **0.03** | 0.62 | 0.06 | 0.72 | 0.17 | 0.40 | **0.03** | 0.47 |
| rs9483670 | 0.63 | 0.48 | 0.78 | 0.72 | 0.74 | 0.74 | 0.81 | 0.40 | 0.27 | 0.79 | 0.57 | 0.29 | 0.62 | 0.74 | 0.48 | 0.55 | 0.76 |
