# Supplementary material for: Adaptive Variation Regulates the Expression of the Human SGK1 Gene in Response to Stress
Source: PLoS Genet. 2009 May 22;5(5):e1000489. doi: 10.1371/journal.pgen.1000489 (PMC2679193; doi:10.1371/journal.pgen.1000489)
Supplement: Table S6 — FST values in Hausa vs. Italians for the SNPs identified by re-sequencing. (0.06 MB DOC) [file pgen.1000489.s007.doc]

Table S6: F_ST_ values in Hausa vs. Italians for the SNPs identified by re-sequencing. In bold, SNP rs9493857

| **Position** | **Region** | **Fst** |
| --- | --- | --- |
| **134572390** | **30Kb upstream** | **0.4029** |
| 134557712 | 20Kb upstream | 0.3125 |
| 134557805 | 20Kb upstream | 0.3125 |
| 134571323 | 30Kb upstream | 0.3075 |
| 134569884 | 30Kb upstream | 0.2899 |
| 134586501 | 50Kb upstream | 0.1787 |
| 134569881 | 30Kb upstream | 0.1697 |
| 134569973 | 30Kb upstream | 0.1483 |
| 134568608 | 30Kb upstream | 0.1385 |
| 134569379 | 30Kb upstream | 0.1323 |
| 134557990 | 20Kb upstream | 0.1264 |
| 134540072 | promoter | 0.1150 |
| 134586154 | 50Kb upstream | 0.0914 |
| 134539932 | promoter | 0.0909 |
| 134572255 | 30Kb upstream | 0.0907 |
| 134570014 | 30Kb upstream | 0.0832 |
| 134583964 | 50Kb upstream | 0.0832 |
| 134583539 | 50Kb upstream | 0.0715 |
| 134586795 | 50Kb upstream | 0.0715 |
| 134535640 | gene | 0.0678 |
| 134558058 | 20Kb upstream | 0.0600 |
| 134568470 | 30Kb upstream | 0.0565 |
| 134586123 | 50Kb upstream | 0.0564 |
| 134539368 | promoter | 0.0537 |
| 134557674 | 20Kb upstream | 0.0416 |
| 134586148 | 50Kb upstream | 0.0359 |
| 134558059 | 20Kb upstream | 0.0313 |
| 134539883 | promoter | 0.0200 |
| 134539977 | promoter | 0.0200 |
| 134571370 | 30Kb upstream | 0.0192 |
| 134601688 | 70Kb upstream | 0.0192 |
| 134601698 | 70Kb upstream | 0.0192 |
| 134568747 | 30Kb upstream | 0.0072 |
| 134569056 | 30Kb upstream | 0.0072 |
| 134569356 | 30Kb upstream | 0.0070 |
| 134601413 | 70Kb upstream | 0.0069 |
| 134602061 | 70Kb upstream | 0.0000 |
| 134602371 | 70Kb upstream | 0.0000 |
| 134537805 | promoter | -0.0050 |
| 134570759 | 30Kb upstream | -0.0080 |
| 134601463 | 70Kb upstream | -0.0130 |
| 134601546 | 70Kb upstream | -0.0130 |
| 134601714 | 70Kb upstream | -0.0130 |
| 134602439 | 70Kb upstream | -0.0130 |
| 134568867 | 30Kb upstream | -0.0160 |
| 134601571 | 70Kb upstream | -0.0170 |
| 134601665 | 70Kb upstream | -0.0170 |
| 134586130 | 50Kb upstream | -0.0180 |
