# Supplementary material for: Adaptive Variation Regulates the Expression of the Human SGK1 Gene in Response to Stress
Source: PLoS Genet. 2009 May 22;5(5):e1000489. doi: 10.1371/journal.pgen.1000489 (PMC2679193; doi:10.1371/journal.pgen.1000489)
Supplement: Table S7 — Summary statistics for the six regions resequenced in Hausa (A), Italians (B) and the overall (C) sample. (0.07 MB DOC) [file pgen.1000489.s008.doc]

Table S7: Summary statistics for the six regions resequenced in Hausa (A), Italians (B) and the overall (C) sample. *p<0.1, **p<0.05, ***p<0.01

A

|  | 70KB UPSTREAM | 50KB UPSTREAM | 30KB UPSTREAM | 20KB UPSTREAM | PROMOTER | GENE | PROMOTER+GENE | TOTAL LENGTH |
| --- | --- | --- | --- | --- | --- | --- | --- | --- |
| Length (Kb) | 1477 | 3727 | 4331 | 1762 | 2740 | 1801 | 4541 | 17039 |
| S | 10 | 9 | 16 | 3 | 2 | 1 | 3 | 41 |
| π (%) | 0.2660 | 0.0410 | 0.1112 | 0.0435 | 0.0056 | 0.0127 | 0.0084 | 0.0652 |
| θ_W_/bp (%) | 0.1740 | 0.0621 | 0.0949 | 0.0438 | 0.0188 | 0.0143 | 0.0170 | 0.0618 |
| Tajima’s D | 1.7009*** | -1.0696* | 0.5909 | -0.0114 | -1.4659* | -0.1782 | -1.2107* | 0.2038 |
| Fu & Li’s D* | 0.3509 | -2.0561 | 0.8271 | 0.9586 | -2.2442 | 0.6028 | -1.4395 | -0.1746 |
| No. of Haplotypes | 7 | 8 | 16 | 4 | 3 | 2 | 4 | 25 |
| Haplotype Diversity | 0.6481 | 0.8016 | 0.9233 | 0.4788 | 0.1402 | 0.1984 | 0.3228 | 0.9894 |
| Fay & Wu’s H | 1.1058 | 0.7992 | 0.0206 | -0.5919 | -0.1477 | -0.1957 | -0.3433 | 1.1852 |
| Fu & Li’s D | 0.3332 | -1.6896 | 0.8799 | 0.9633 | -2.3368 | 0.5881 | -1.5359 | -0.0905 |

B

|  | 70KB UPSTREAM | 50KB UPSTREAM | 30KB UPSTREAM | 20KB UPSTREAM | PROMOTER | GENE | PROMOTER+GENE | TOTAL LENGTH |
| --- | --- | --- | --- | --- | --- | --- | --- | --- |
| Length (Kb) | 1477 | 3727 | 4331 | 1762 | 2740 | 1801 | 4541 | 17039 |
| S | 10 | 7 | 10 | 3 | 3 | 1 | 4 | 34 |
| π (%) | 0.2749 | 0.0293 | 0.0672 | 0.0602 | 0.0288 | 0.0269 | 0.0280 | 0.0588 |
| θ_W_/bp (%) | 0.1740 | 0.0483 | 0.0593 | 0.0438 | 0.0281 | 0.0143 | 0.0226 | 0.0513 |
| Tajima’s D | 1.8665** | -1.1823** | 0.4247 | 0.9049 | 0.0541 | 1.4141 | 0.6243 | 0.5448 |
| Fu & Li’s D* | 1.4063 | -1.4619 | 0.3509 | -0.2405 | 0.9586 | 0.6028 | 1.0659 | 0.3962 |
| No. of Haplotypes | 6 | 5 | 16 | 3 | 4 | 2 | 5 | 24 |
| Haplotype Diversity | 0.6402 | 0.5820 | 0.9021 | 0.5212 | 0.3228 | 0.4233 | 0.6190 | 0.9735 |
| Fay & Wu’s H | 0.9894 | 1.1581 | 3.4332 | -0.4431 | -0.6509 | -0.2078 | -0.8587 | 4.2434 |
| Fu & Li’s D | 1.5030 | -1.6362 | 0.3332 | -0.2863 | 0.9633 | 0.5881 | 1.0841 | 0.3971 |

C

|  | 70KB UPSTREAM | 50KB UPSTREAM | 30KB UPSTREAM | 20KB UPSTREAM | PROMOTER | GENE | PROMOTER+GENE | TOTAL LENGTH |
| --- | --- | --- | --- | --- | --- | --- | --- | --- |
| Length (Kb) | 1477 | 3727 | 4331 | 1762 | 2740 | 1801 | 4541 | 17039 |
| S | 12 | 12 | 20 | 6 | 5 | 1 | 6 | 56 |
| π (%) | 0.2675 | 0.0360 | 0.1042 | 0.0613 | 0.0176 | 0.0207 | 0.0188 | 0.0665 |
| θ_W_/bp (%) | 0.1769 | 0.0701 | 0.1005 | 0.0741 | 0.0397 | 0.0121 | 0.0288 | 0.0715 |
| Tajima’s D | 1.4862 | -1.4129 | 0.1147 | -0.4320 | -1.321 | 0.9251 | -0.8634 | -0.2410 |
| Fu & Li’s D* | 0.3665 | -2.4109 | 0.1658 | 0.2892 | -0.8744 | 0.5336 | -0.5874 | -0.5900 |
| No. of Haplotypes | 10 | 10 | 29 | 6 | 6 | 2 | 7 | 49 |
| Haplotype Diversity | 0.6409 | 0.7045 | 0.9506 | 0.5338 | 0.2344 | 0.3214 | 0.4877 | 0.9909 |
| Fay & Wu’s H | 0.9714 | 0.8682 | 0.4092 | -0.8881 | -0.4484 | -0.2551 | -0.7035 | 0.7532 |
| Fu & Li’s D | 0.3623 | -2.0045 | 0.1476 | 0.2787 | -0.9233 | 0.5275 | -0.6317 | -0.5263 |
