# Supplementary material for: Adaptive Variation Regulates the Expression of the Human SGK1 Gene in Response to Stress
Source: PLoS Genet. 2009 May 22;5(5):e1000489. doi: 10.1371/journal.pgen.1000489 (PMC2679193; doi:10.1371/journal.pgen.1000489)
Supplement: Table S8 — Association p values for rs9493857 and proxy SNPs from publicly available datasets. (0.08 MB DOC) [file pgen.1000489.s009.doc]

Table S8: Association p values for rs9493857 and proxy SNPs from publicly available datasets. * Study accession is reported in parentheses

|  |  | SNP (r^2^ with rs9493857) | | | |
| --- | --- | --- | --- | --- | --- |
| Source/Study | Phenotype | rs9493857 (1) | rs4896028 (0.811) | rs13203172 (0.729) | rs1009840 (0.616) |
| British 1958 Birth Cohort | log10 IgE | NA | >0.1 | >0.1 | >0.1 |
|  | systolic BP | NA | >0.1 | >0.1 | >0.1 |
|  | adult BMI | NA | 0.027 | >0.1 | >0.1 |
|  | adult height | NA | >0.1 | >0.1 | >0.1 |
|  | FEV1 | NA | >0.1 | >0.1 | >0.1 |
|  | 4 kHz threshold | NA | >0.1 | >0.1 | >0.1 |
|  | cholesterol | NA | >0.1 | >0.1 | >0.1 |
|  | log10 HbA1c | NA | >0.1 | >0.1 | 0.038 |
|  | log10 fibrinogen | NA | >0.1 | >0.1 | >0.1 |
|  | birth weight | NA | >0.1 | >0.1 | >0.1 |
| WTCCC | T2D | NA | NA | >0.1 | >0.1 |
|  | T1D | NA | NA | >0.1 | >0.1 |
|  | RA | NA | NA | >0.1 | >0.1 |
|  | HT | NA | NA | >0.1 | >0.1 |
|  | CD | NA | NA | >0.1 | 0.06 |
|  | CAD | NA | NA | >0.1 | >0.1 |
|  | BD | NA | NA | >0.1 | >0.1 |
| Broad Institute, Diabetes Genetics Initiative | T2D | NA | NA | NA | >0.1 |
| dbGaP* | Neuroblastoma (phs000124.v1.p1) | NA | >0.01 | NA | >0.01 |
|  | Diabetic nephropathy in T1D (phs000018.v1.p1) | NA | NA | NA | >0.01 |
|  | Psoriasis (phs000019.v1.p1) | NA | >0.01 | NA | >0.01 |
|  | ADHD (phs000016.v1.p1) | NA | >0.01 | NA | 0.01-0.001 |
|  | Systemic Lupus Erithematosus (phs000122.v1.p1) | NA | >0.01 | NA | 0.01-0.001 |
|  | Parkinson disease (phs000048.v1.p1) | >0.01 | NA | NA | >0.01 |
|  | T2D (phs000100.v1.p1) | NA | >0.01 | NA | >0.01 |
|  | YKL-40level (phs000123.v1.p1) | NA | NA | NA | >0.01 |
|  | Multiple Sclerosis (phs000139.v1.p1) | NA | NA | NA | >0.01 |
|  | Parkinson disease (phs000089.v1.p1) | NA | >0.01 | NA | >0.01 |
|  | Amyotrophic Lateral Sclerosis (phs000101.v1.p1) | NA | >0.01 | NA | >0.01 |
|  | Ischemic stroke (phs000102.v1.p1) | NA | >0.01 | NA | >0.01 |
|  | Amyotrophic Lateral Sclerosis (phs000127.v1.p1) | NA | >0.01 | NA | >0.01 |
|  | Crohn's disease (phs000130.v1.p1) | NA | >0.01 | NA | >0.01 |
